# Self-supervised machine learning for live cell imagery segmentation

**DOI:** 10.1038/s42003-022-04117-x

**Published:** 2022-11-02

**Authors:** Michael C. Robitaille, Jeff M. Byers, Joseph A. Christodoulides, Marc P. Raphael

**Affiliations:** Materials Science and Technology Division, U.S. Naval Research Laboratory, Washington, DC USA

**Keywords:** Cellular imaging, Machine learning, Optical imaging

## Abstract

Segmenting single cells is a necessary process for extracting quantitative data from biological microscopy imagery. The past decade has seen the advent of machine learning (ML) methods to aid in this process, the overwhelming majority of which fall under supervised learning (SL) which requires vast libraries of pre-processed, human-annotated labels to train the ML algorithms. Such SL pre-processing is labor intensive, can introduce bias, varies between end-users, and has yet to be shown capable of robust models to be effectively utilized throughout the greater cell biology community. Here, to address this pre-processing problem, we offer a self-supervised learning (SSL) approach that utilizes cellular motion between consecutive images to self-train a ML classifier, enabling cell and background segmentation without the need for adjustable parameters or curated imagery. By leveraging motion, we achieve accurate segmentation that trains itself directly on end-user data, is independent of optical modality, outperforms contemporary SL methods, and does so in a completely automated fashion—thus eliminating end-user variability and bias. To the best of our knowledge, this SSL algorithm represents a first of its kind effort and has appealing features that make it an ideal segmentation tool candidate for the broader cell biology research community.

## Abstract (web summary)

A self-supervised learning approach uses cellular motion between consecutive images to self-train a machine learning classifier for cell segmentation.

## Introduction

The information stored in time-lapse live cell microscopy imagery is of paramount importance to cell biology. In particular, two-dimensional (2D) cell cultures and experiments are widespread in both academic and industrial research, regulatory processes, and commercial pipelines. Thus, there is a well-established need for quantitative bioimage analysis tools, often in the form of cell segmentation. Over the past decade, machine learning has emerged as a powerful method for cell segmentation^1–3^. Machine learning offers a framework, supervised learning (SL), that combines the data with human annotated labels to form a classifier model for identifying features of interest. In particular, Artificial Neural Networks (ANNs) have been a popular SL technique in bioimage analysis in recent years, as they typically outperform standard image processing pipelines^3,4^.

A major drawback of machine learning is that it is data hungry. In particular, ANNs typically require an immense amount of labeled data for good performance on complex data sets, in a step typically referred to as data pre-processing. For example, standard computer vision training libraries like Microsoft’s COCO contains over 1 million label objects to adequately train ANNs^5^. The problem with this approach is that imagery in cell biology is incredibly diverse when compared to the imagery typical for internet-related computer vision problems (*i.e*. animal recognition). Consequentially, there are numerous large-scale efforts to create ever larger training libraries to address this need, such as the recently curated EVICAN^6^ (26,000 labeled objects), CellPose^7^ (70,000 labeled objects), and LIVEcell^8^ (1.6 million labeled objects) libraries, in hopes of achieving robust models that can simply be utilized by the larger cell biology research community. However, underlying all SL, including ANNs, is the fact that models will only perform reliably on data similar to those used during training^9^. This “big library” approach is no match for the sheer breadth of cell types, optical modalities, microscope configurations, 2D and 3D extracellular environments and customized experimental conditions which embody cell microscopy—all of which are continuously evolving. The common motto in machine learning, “when in doubt, retrain”, is a clear testament to this fact, yet model training is far from trivial and a notoriously labor-intensive task^10^, often at the expense of the end-user.

While the field can keep pursuing a “bigger is better” philosophy with regards to training libraries, there is a growing realization that subjective elements enter machine learning models via data labeling^11–14^, causing biases to be effectively baked into the extracted data by the training process in ways that are poorly defined and difficult to determine. Furthermore, due to the opaque nature of model weights and potential overfitting when spanning such a large parameter space, the efficacy of large libraries is still an active question. To increase machine learning accessibility to the broader cell biology community an alternative approach is required that does not rely upon “bigger is better” training strategies. One alternative strategy is self-supervised learning (SSL). SSL leverages some underlying features in the data itself as a means of supervision, or labeling data, and is promising as it can learn directly from the end-user’s own data—eliminating the need for labor-intensive curated libraries and the biases contained within. For time-lapse live cell imagery, there is a prominent data structure that can be used to self-label data, regardless of what cell type, optical modality or otherwise experimental set up used: motion.

Here, we show that optical flow between consecutive images can be used as a means to self-label data for cell segmentation (zero shot learning). We then construct an algorithm that trains itself with this self-labeled data to classify cells versus background, and can do so in a completely automated fashion. We validate our algorithm on a variety of live cell imagery, spanning five optical modalities (both fluorescent and tag-free) and extensively different experimental set ups to show its applicability to the broader cell biology research community. By leveraging motion inherent in time-lapse live cell imagery, we show that (1) SSL eliminates human intervention; there is no need to build ever expanding training libraries, (2) SSL allows for complete automation, an important step forward in eliminating bias and producing reproducible machine learning efforts in cell biology, and (3) SSL has no dependencies on particular cell type, optical modality, or experimental environment. Together, all three of these advantages translate into a robust, reproducible and user-accessible cell segmentation tool.

We show that by leveraging motion as a means of self-supervision directly from the data to be analyzed, we outperform state-of-the-art ANN generalist models with curated libraries of over tens of thousands of labels. To the best of our knowledge, this work represents the first-of-its-kind completely automated general cell segmentation algorithm. More importantly, our SSL approach directly addresses the pre-processing problem in machine learning, and offers a path forward to increase unbiased machine learning accessibility for cell biology laboratories.

## Results

### Self-labeling algorithm overview

To replace manual and supervised learning approaches for segmenting cells with a self-supervised algorithm, we took advantage of the one phenotypic feature which is always present in live cell microscopy: *motion*. The ever present dynamics captured by live cell microscopy make it ideal for applying optical flow (OF) algorithms designed to identify the variation or ‘flow’ of image features from frame to frame. Optical flow algorithms are founded upon the assumption that two images can be related by a spatial shifting of their pixel values. The methods used to calculate these displacements are matched with various imaging goals, such as motion detection, guiding autonomous vehicles, stabilizing imagery taken from moving platforms, aligning medical imagery or, in the case of this study, cell motion segmentation^15^. To account for the facts that cells are highly deformable and that live cell imagery typically incorporates jitter from scanning stage motion, a multi-resolution Farneback Displacement^16^ (FD) optical flow algorithm was employed (Supplementary Note 3).

**Fig. 1:**
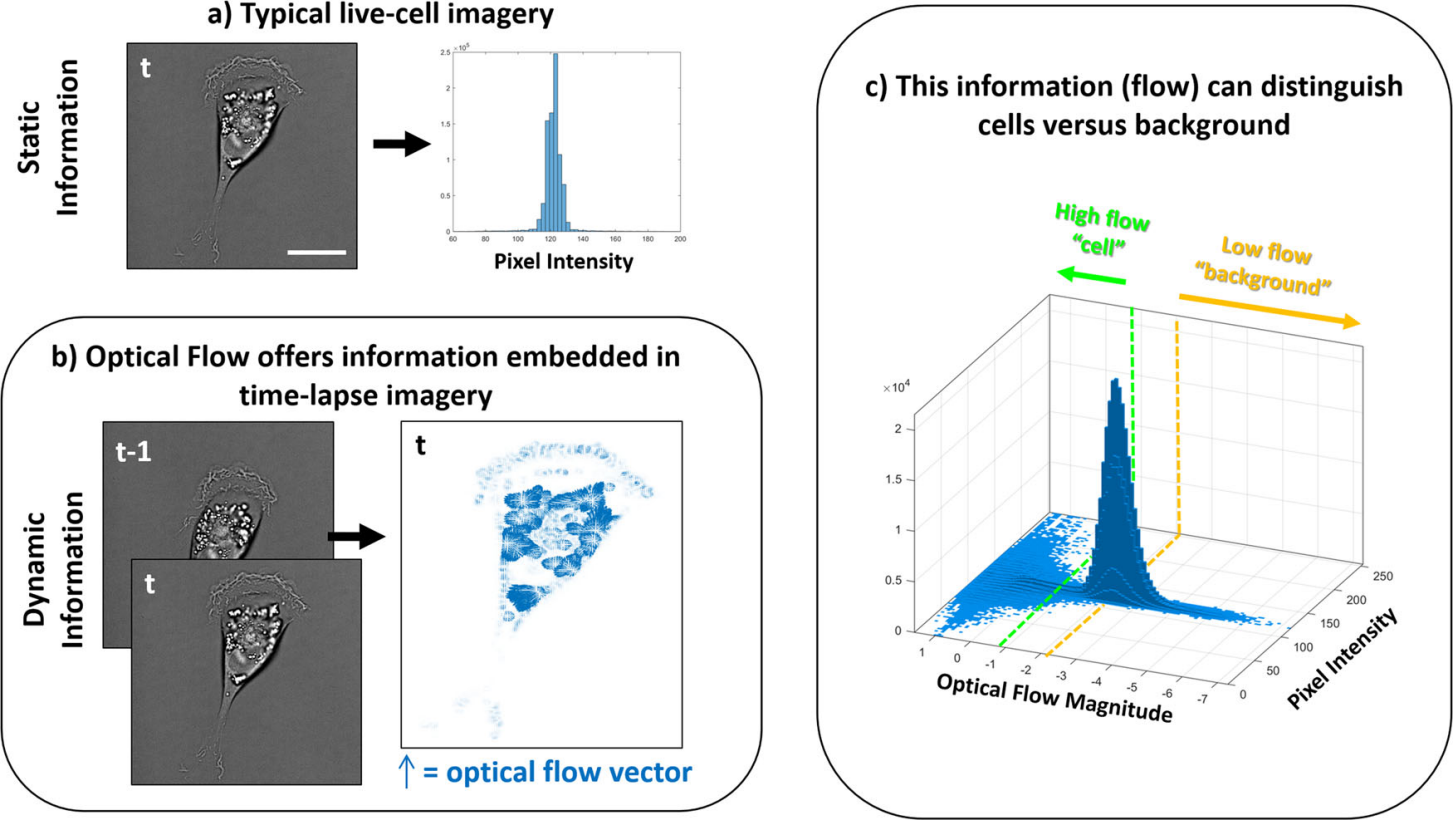
Overview of the Farneback displacement (FD) self-labeling strategy. **a** The vast majority of cell segmentation techniques utilize single image frames and the static information contained within as means to distinguish ‘cell’ from ‘background’, oftentimes represented in a histogram. The self-supervised algorithm utilizes optical flow as a means to self-label pixels in an automated fashion. **b** Due to the prevalence of intracellular dynamics in time-lapse live cell imagery, FD can be calculated for each pair of consecutive images $\left(t-1,{t}\right)$. The FD can then be represented as vectors associated with each pixel (right). **c** The magnitude of the FD then offers a means to distinguish cells from their background, as shown in the bivariate histogram which co-plots the pixel intensity of a single image at *t* to the FD vector magnitudes calculated between consecutive images $\left(t-1,{t}\right)$. Pixels with the highest displacements can be automatically labeled ‘cell’ (left of the green dashed line) and those with the lowest can be labeled ‘background’ (right of the yellow dashed line). Pixels that do not meet either criteria remain unlabeled, while the self-labeled pixels are used to create a training data set for classification. Time increment: 600 s, scale bar = 20 µm.

Our approach to self-supervised learning and automated model generation begins with using FD to automate the training process (Fig. 1). Typical segmentation strategies involve utilizing static information in a single image at time frame (*t*), which can have difficulty distinguishing ‘cell’ from ‘background’ pixels in a generalizable manner (Fig. 1a). In contrast, our approach begins with a FD calculation based on images from consecutive time frames (*t*-1, *t*). This enables us to leverage the ubiquitous nature of intracellular motion and build a dynamics-based feature vector: pixels with the highest displacements are automatically labeled as ‘cell’ pixels, those with the lowest displacement are automatically labeled as ‘background’ pixels, and those that do not fit either category remain unlabeled (Fig. 1b, c). We note that this automatic self-labeling is broadly applicable in that it is not dependent on principles of any specific optical modality, cell type, or phenotype. The robustness of applying optical flow to self-supervised learning stems from the fact that the algorithm detects intracellular motion as well as motion due to overall cell migration. As a result, motion of internal structural components such as organelles and membrane fluctuations contribute to the classification process, and if applied to fluorescently tagged cells, fluorescently labeled molecules contribute as well.

The FD-based self-labeling approach outputs a set of ‘cell’ and ‘background’ labeled pixels which are then used to generate additional entropy and gradient feature vectors at each time point. These static feature vectors are then used to train and generate a classifier model which, in the final step, is applied to all pixels in the image for cell segmentation.

**Fig. 2:**
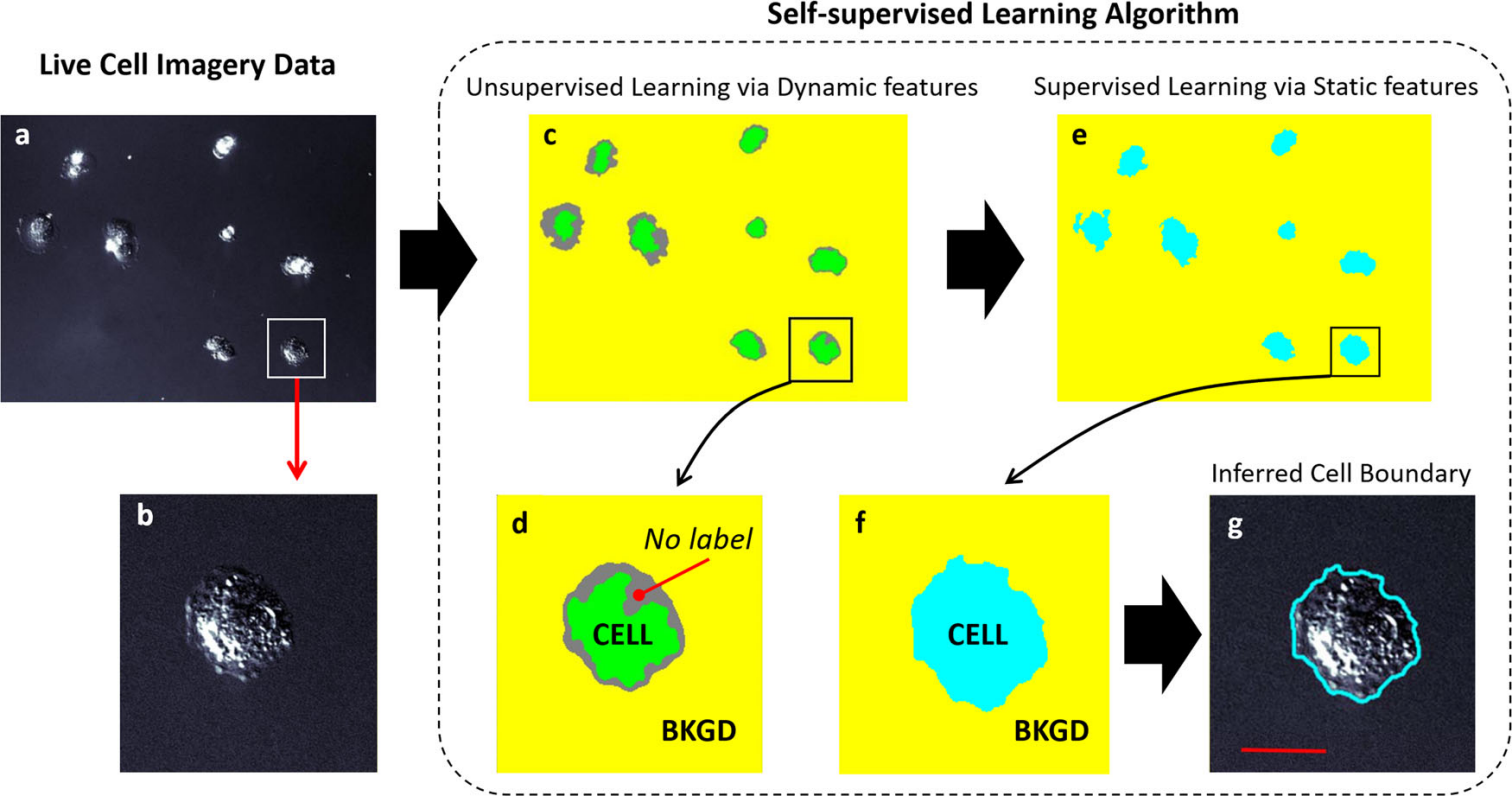
Overview of the automated self-supervised learning algorithm. **a** The contrast enhanced DIC image of several and **b** a single highlighted MDA-MB-231 cell illustrates the range of intensities inherent within the cells. (20X objective). **c**, **d** Unsupervised learning via FD: high threshold FD is used to select only those pixels exhibiting the highest displacement magnitudes and labels them as ‘cell’ (green pixels). Similarly, low threshold FD is used to identify pixels with a much wider range of displacement magnitudes than the high flow regime. The lowest displacement magnitude pixels are labeled ‘background’ (yellow pixels). Pixels that exhibit FD in between these regimes remain unlabeled (gray pixels). **e**, **f** Supervised learning via self-labeled training data. The self-labeled pixels (green and yellow) are then used to generate static feature vectors, which are in turn used to train the classifier model. **g** The blue outline is the resulting segmentation which outlines all pixels classified by the FD trained model as ‘cell’ and is also overlaid on the image in **b**. This process is repeated at every time step, thereby using the most recent imagery to update the training data. Scale bar: 25 µm (20X objective, time increment: 300 s).

The complete self-supervised approach to segmentation based on FD self-labeling is illustrated in Fig. 2 using time lapse DIC imagery of multiple (top) and a single highlighted (bottom) MDA-MB-231 cell. From the raw imagery (Fig. 2a, b), many portions of individual cells appear to blend in with the background. However, when the FD self-labeling strategy is applied, the algorithm automatically identifies pixels with high displacement magnitude, highlighted as green pixels (Fig. 2c, d), which are selected as having the highest probability of correctly being labeled ‘cell’. This identification may be due to overall cell motion or intracellular dynamics as highlighted by the blue optical flow vectors in Fig. 1b. To automatically label the background, the algorithm over segments, that is, a liberal (low) FD threshold is employed which captures motion from not only the cell but also from nearby background pixels as well. The algorithm sets these pixel values to zero and labels the remaining pixels as ‘background’ (Fig. 2c, d yellow pixels). Once labeled ‘cell’ or ‘background’ in this unsupervised manner by FD (dynamic features from image pair $\left(t-1,{t}\right)$), entropy and gradient feature vectors (static features from image at *t*) are generated for each of these training pixels using their local neighborhood of pixels (Supplementary Note 1, Fig S1). These additional feature vectors are then used to train and generate a Naïve Bayesian classifier model which is applied to the entire image in a pixel-wise fashion. The information gained from the entropy and gradient feature vectors enables pixels which were left unlabeled in the FD training steps (Fig. 2c, d gray pixels) to be classified. The contrast enhanced image (Fig. 2b) and model-generated segmentation (Fig. 2e, f, teal pixels) show that the algorithm is able to segment the cell with high fidelity (DIC image/segmented boundary overlay, Fig. 2g). Importantly, this labeling, training and classifying procedure occurs recursively on each successive pair of $\left(t-1,{t}\right)$images, enabling the classifier model to adapt to changing backgrounds and phenotypes. By using FD to label the highest displacement pixels as ‘cells’ and lowest displacement pixels as ‘background’, the labeling process has become automated (or ‘self-supervised’) and no manual inputs or curated training libraries are needed.

For extremely low contrast imagery there can be too few training pixels labeled ‘cell’ for robust segmentation to occur given the initial FD threshold setting. In such cases, the algorithm calculates the entropy associated with ‘cell’ pixels and iteratively reduces the FD threshold until the associated ‘cell’ entropy feature vector is well distinguished from that of the ‘background’ entropy feature vector.

### Algorithm evaluation

**Fig. 3:**
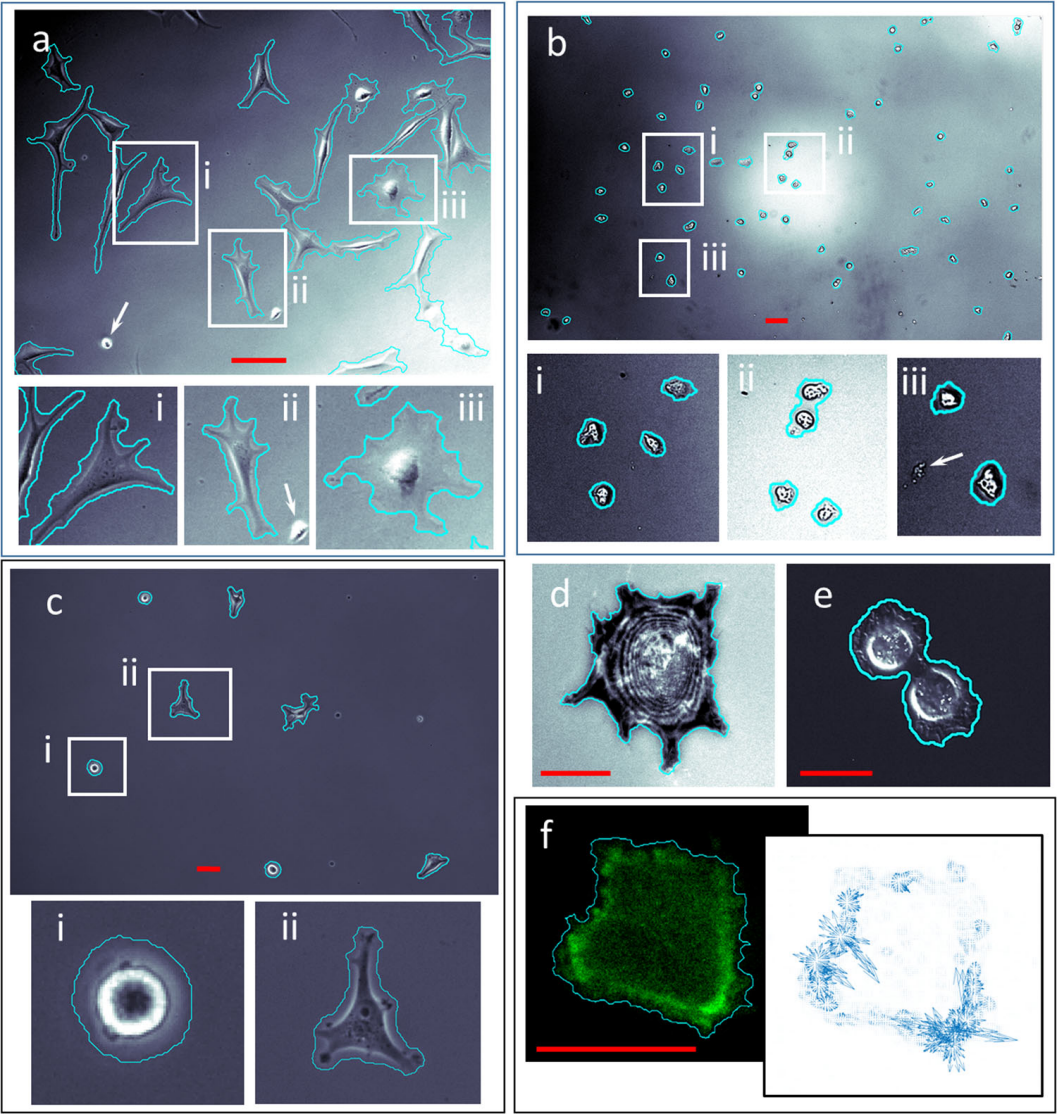
Self-supervised segmentation for a range of cell types, microscope modalities, time resolutions and magnifications. **a** phase contrast of Hs27 fibroblasts (10X objective, time increment: 1200 s) **b** transmitted light of Dictyostelium (10X objective, time increment: 60 s) **c** phase contrast of MDA-MB-231 (10X objective, time increment: 600 s) **d** IRM image of a single Hs27 cell (40X objective, time increment: 600 s). **e** DIC image of MDA-MB-231 cells (20X objective, time increment: 120 s) **f** fluorescence image of a single lifeAct (GFP-actin conjugate) transfected A549 cell (pseudo-colored) with the associated FD vector plot (100X objective, time increment: 10 s). Insets i, ii, iii highlight boxed image regions. White arrows point to examples of debris that was correctly labeled ‘background’ due either to lack of motion or automated size filtering. Images have been contrast enhanced to highlight low contrast features and background inhomogeneities. DIC image **e** was additionally enhanced with a sharpen filter to highlight interference induced shadowing of cell features. Scale bars: **a**, **b**, **c**: 50 µm; **d**, **e**: 25 µm; **f**: 10 µm.

A central theme of this work is that machine learning approaches which require supervised training can be time consuming, subjective, and ultimately ineffective. The training process is widely recognized as the most time consuming aspect of machine learning approaches. Due to the opaque nature of many machine learning algorithms, and in particular deep learning techniques, the reasons behind the success or failure of a training data set is often not clear to the end user. Hence, the process is one of trial and error, requiring retraining if the model’s performance is not deemed adequate^14^. To evaluate segmentation by our self-supervised approach, we compiled a diverse imagery data set (Fig. 3, Supplementary Note 2, Table S1). For comparison against a supervised learning approach with a curated training library, we chose the recent popular artificial neural network CellPose^7^, which consists of a model pre-trained on 70,000 manually annotated objects spanning multiple optical modalities, cell types, and objects. Like our approach, CellPose is trained to be a generalist model applied to the wider cell biology research community, and furthermore has the option for automated analysis, thus making it an ideal algorithm for comparison. The F_1_-score metric was calculated to evaluate the quality of segmentation done by each method on each data set. For each data set, cells are manually segmented to serve as the ground truth against the segmentation for each method. The true positives (TP), false positives (FP), and false negatives (FN) of each method are calculated in a pixel-wise fashion. The F_1_-score is then defined as:

1 $${F}_{1}=\frac{{TP}}{{TP}+\frac{1}{2}({FN}+{FP})}$$

The Fig. 3 imagery shows the generality of this approach and also demonstrates how the self-supervised algorithm additionally automates commonly required manual inputs such as size filtering and hole filling. The segmented cells were processed from imagery acquired from a range of cell types, imaging modalities, magnifications and time increments (Supplementary Note 2, Table S1). The FD algorithm enabled a straightforward approach to automated size filtering which is a common user adjustable parameter in supervised machine learning approaches. To accomplish this, a stand-alone application of FD was applied to the imagery which lacked the added steps of self-tuning and model building described above. While some cell features are missed, this simpler, faster approach was found to be more than precise enough to estimate average cell size and to exclude much smaller objects, thus automating the size filtering process. Because extraneous debris often lacked the motion of the live cells, this debris was also automatically labeled as background by the FD algorithm. Figure 3a, b demonstrate the self-supervised code’s ability to size filter, while also adapting to cell types of differing sizes, by comparing the segmentation of human fibroblasts (10X, phase contrast) to those of the much smaller Dictyostelium ameboid cells (10X, transmitted light), respectively. Extraneous debris features in the Hs27 imagery (Fig. 3a, white arrows) are correctly identified as ‘background’, even though similar in size and intensity to the Dictyostelium cells of Fig. 3b. The background inhomogeneities observed in Fig. 3a, b, which could potentially be mislabeled as ‘cell’, are correctly identified because they remain relatively constant from frame $t-1$ to frame $t$. The segmentation results of the MDA-MB-231 cells (10X, phase contrast) in Fig. 3c illustrates the algorithm’s ability to adapt to a wide range of phenotypes, from rounded Fig. 3c(i) to spread Fig. 3c(ii), which is enabled without need for user input by continuously retraining the model on consecutive image pairs.

The algorithm works robustly for a range of optical modalities and magnifications as shown in Fig. 3d–f. Figure 3d, e are segmentation results from IRM imagery (40X, Hs27 cell) and DIC imagery (20X, MDA-MB-231). As a fluorescence imaging example, a self-supervised segmentation of a GFP-actin tagged A549 cell at 100X magnification is shown in Fig. 3f. As an additional option, FD can be applied not only as an algorithm labeling element, but also a measurement tool, as shown in the Fig. 3f vector plot. The plotted FD vectors (blue) display the magnitude and direction of the measured GFP tagged actin flow between frames. Such measurements have been shown to be useful for quantifying intracellular protein and calcium signaling dynamics^17–19^.

Hole filling, another often required manual input for image processing-based and machine learning algorithms, has also been automated by this approach. Common examples of when hole filling input is required include fluorescent tags that do not penetrate the nucleus or, for tag-free microscopy modes such as phase contrast, large spread cells in which the algorithm has a difficult time associating the interference enhanced cell edges with the enclosed lamellipodia. We found that motion within cells was ubiquitously detected by FD, regardless of imaging modality or whether imaging the cell membrane, nucleus or cytoplasm. Because motion detection was far more common than not for a given pixel within an area labeled ‘cell’, a fixed morphological blurring tool (circular with a radius of 5 pixels) was found to robustly hole fill regardless of cell type or microscope configuration. The calculated cell area was found to be invariant for a range of blurring tool radii (Supplementary Note 4, Fig. S3). In all cases, the use of optical flow to identify motion and the 5 pixel radius blurring tool was sufficient to correctly fill in the cell.

**Fig. 4:**
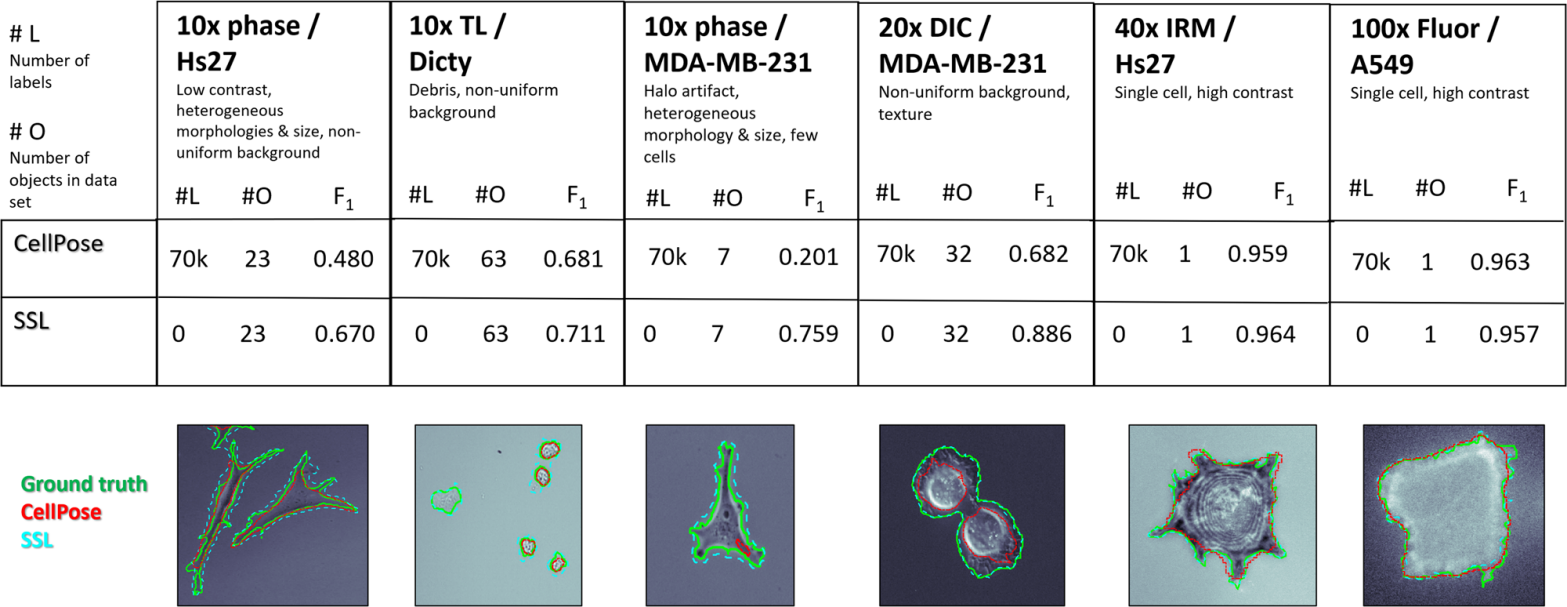
Segmentation evaluation of Self-Supervised Machine Learning (SSL) and CellPose on the data sets used for validation in this study via F_1_-scores. The top row includes the name of the data set annotated by magnification, optical modality, cell type and brief description of the imagery characteristics. #L stands for the number of annotated labels used for model training, and #O stands for the number of objects to be segmented by the model within a given data set. *CellPose has a single parameter, a size filter, that can be automatically estimated, however, for some of the data sets the best segmentation was found by manually tuning this size filter. The figures below show the ground truth (green-solid lines), SSL (cyan-large dashes), and CellPose (red-small dashes) outlines overlaid on the final image of the data set.

A comparison of our SSL approach versus CellPose is shown in Fig. 4 via F_1_-scores, with a brief description of each data set given at the top, including how many annotated labels were used in each model and applied to how many objects (cells) within each data set. CellPose^7^ is a relatively new supervised learning framework that is trained to identify intensity gradients and is based on a general U-Net neural network architecture^20^. To achieve this, the authors have taken considerable resources to train their model off of 70,000 manually annotated objects, including both fluorescent and tag-free images, and this was directly applied to the data sets representing both common and more specialized modes of microscopy in cell biology. In contrast, our SSL trained on the data sets itself without any need from human input (#L = 0). Figure 4 shows that SSL performed well across data sets, achieving F1-scores from ~0.7–0.9 indicating robust performance across diverse live-cell imagery. SSL outperformed CellPose in four of the data sets used for validation in this study that were largely lower magnification and multi-cellular data sets. In the two remaining data sets that were higher magnification of single-cells, the performance of each method was statistically equivalent. Details of CellPose segmentation on the data sets are shown in the Supplementary Note 5, Figs. S4–S9.

## Discussion

The past decade has seen significant efforts and improvements to the application of machine learning (ML), and in particular supervised learning techniques to cell segmentation. However, the well-defined nature of the supervised learning framework can hide many assumptions about the relationship between the data and corresponding labels – that is, that humans are actively supervising the labeling and training processes during pre-processing steps. Despite great improvements making ML more accessible^7,10,21^, this pre-processing requirement of supervised learning is one of the reasons ML has yet to widely transition from computer scientists to the wider cell biology research community—it hinders efficiency and poses a serious challenge for ensuring reproducibility in bioimage analysis. Thus, it is crucial that the field strive for the high standard of *general* strategies that can segment cell imagery from any research group *without input from the user*. Indeed, this was the scope of the 2018 Data Science Bowl, which set out to establish a completely automated algorithm for segmentation of fluorescently tagged nuclei^22^. Our SSL approach represents a natural extension of this line of thought, broadening the automation to entire cell segmentation in time-lapse imagery, with the goal of this work being to create a broadly applicable ML strategy (1) without the need of input or configuration from the end-user and (2) without the need of any data pre-processing (i.e. manual labeling).

Our SSL approach accomplishes this by building a continuously evolving model that retrains itself on each new image via the Farneback displacement (FD), a dynamic feature vector imbedded in the data structure of time lapse imagery. From the FD, additional static feature vectors can easily be generated for model training. In this work we primarily studied two such static feature vectors—gradient and entropy—but the code is modular in this regard and there are many additional image features that can be added based on the application. While optical flow has been used previously for biological imagery, it has largely been in the context of spatio-temporal characterization of fluorescently tagged proteins^23,24^, and much less seldom applied to cell segmentation in a general way^15^. Here, we show that the evolution of cellular dynamics captured by FD can be leveraged as a powerful means to continuously self-train ML algorithms. One consequence of this continual training is that cell features or background illumination, which inevitably vary over time, do not need to be manually anticipated a priori as the same imagery to be segmented is also used for training.

Due to the exponential growth of ML applied to the life sciences in recent years^25^, there has been more attention on establishing and adopting best practices to ensure reproducibility of ML applied to bioimage analysis. Often the discussions are centered around issues like reporting documentation of training data sets, data augmentation, and hyperparameters used, to name a few, in an attempt to achieve transparency in how ML models were trained and applied^14^. The unencumbered approach of our SSL strategy outlined here succinctly sidesteps many of these issues due to the fact that it is completely automated—easily achieving the recently established “gold standard” for reproducibility of ML in lifesciences^26^ as long as end-users simply make their data available. However, even this gold standard does not address the concerns about biases built into the networks themselves during both the selection and annotation of training data in pre-processing steps^11–14^. For instance, the authors of LIVEcell library took incredible care to structure and manage the annotation of their library to avoid bias^8^ due to the industrial and regulatory application of their work. However, these cautionary steps are rarely applied in research-based ML training libraries due to the sheer cost and resources associated with implementing them. Again, the automation enabled by SSL largely sidesteps concerns of bias in data labeling/pre-processing and offers an appealing strategy to ensure reproducibility in ML efforts on a wider scale.

In general, model training is a significant barrier into both the accessibility^10^ and reproducibility^14^ of machine learning in cell biology. Once trained, models can be applied effectively on data that is similar to that which it was trained on initially. However, the use of pre-trained models on new and distinct data sets, or transfer learning, is a current hurdle ML, and in particular SL approaches struggle with. The relatively poor performance of CellPose on our validation data sets, despite the use of a large and diverse training library, are a testament to how sensitive the performance of state-of-the-art ANNs is on the choice and curation of training data sets. We note that CellPose is not unique in this regards, but rather this phenomenon is systemic in SL approaches and in particular ANNs^27^.

The algorithm presented here is not of overly-sophisticated architecture, and thus does not require the intense computational power/infrastructure common for many ML pipelines^10^. Quite the opposite, this code was validated only on laptops and could achieve acceptable processing times when using this algorithm on high-resolution microscopy data. For instance, a pair of 1216 × 1920 8-bit images can be self-segmented in ~7 s on the mid-range laptops we used for testing. This helps make our SSL algorithm accessible to common cell biology laboratories, which are largely focused around windows-based microscopy systems. In constructing our algorithm, we initially explored classifiers such as random forests, SVM, and K-Nearest Neighbor. However, the Naïve Bayes classifier was chosen as a flexible and effective option, as they are known to have good bias-variance tradeoff because of their simplistic assumption of feature independence, and was found to perform robustly in the context of cell segmentation outlined here.

The SSL algorithm presented does have limitations. First and perhaps most obvious, it can only be applied to live-cell imagery. Second, due to its nature of self-labeling via optical flow, it requires a stable experimental set up in order to correctly distinguish cells from their background—if the microscope stage is drifting laterally or the focus is drifting, the assumption that only cells are moving relative to a stable background are invalid. In our experiments we found that today’s commercially available live cell microscopes were more than stable enough to meet this criteria and, when not, auto alignment software (such as that included with ImageJ) could be easily incorporated. In its current form, the software is designed for semantic segmentation only and not instance segmentation (i.e. separating touching cells). However, the code is designed to be modular, and future work will focus on adding a de-clumping techniques, such as watershed methods, to the binary mask generated by SSL.

To the best of our knowledge, this work represents a first-of-its-kind effort for automated cell segmentation that can be applied across cell types, optical modalities, or otherwise experimental set ups in cell biology (e.g. from different laboratories). The crux of our approach is to utilize optical flow, specifically a Farnebeck Displacement (FD), between consecutive images of time-lapse live cell imagery as a means to self-label training data for a model that distinguishes cells from their background. This self-supervised strategy enables complete automation—drastically reducing the labor of supervised learning techniques, eliminating sources of bias from the curation and labeling of training data, and overall represents a step to both increase accessibility of ML to cell biology labs and introduce a strategy that aids reproducibility in ML.

## Methods

### Cell culture and microscopy

All mammalian cells were cultured in DMEM (ATCC, #30-2002) supplemented with 10% fetal bovine serum (ATCC, #30-2020) at 37 °C and 5% CO_2_, and all imaging of mammalian cells was conducted under serum free conditions (DMEM alone). Hs27 fibroblasts (ATCC, #CRL 1634) were imaged on planar sections of quartz contact guidance chips as previously described^28^. MDA-MB-231 cells (ATCC #HTB-26) were imaged on glass bottomed well plates coated with 25 µg/mL Fibronectin (Gibco #33016015) or functionalized gold coated coverslips as previously described^29^. A549 cells (ATCC #CCL-185) were imaged on planar sections of quartz nanostructured chips as previously described^30^. The Dictyostelium cells were wild type AX2 strain and generously gifted from the Devreotes laboratory of Johns Hopkins University, were cultured axenically in HL5 at 22 °C, and imaged on glass bottom well plates as previously described^31^. Microscopy details for each cell type including microscopy mode, magnification, numerical aperture, camera and wait time between images are listed in Supplementary Note 2.

### Statistics and reproducibility

Each segmented image was produced from two consecutive images in the time series (*N* = 2). The self-supervised methodology is inherently blinded and reproducible because it does not rely upon curated data sets or user-determined parameter settings, but rather trains itself from the image data.

### Reporting summary

Further information on research design is available in the Nature Research Reporting Summary linked to this article.

## Supplementary information

Supplementary Information

Description of Additional Supplementary Files

Supplementary Data

Supplementary Software 1

Supplementary Software 2

Reporting Summary

## Data Availability

Images evaluated in Figs. 3, 4 are available as TIFF files in the Supplementary Data and are also included in the code packages available at Zenodo^32^.

## Code availability

The SSL application is available as a stand-alone GUI download for Windows, Mac and Linux operating systems and as a separate SSL Matlab source code package. Both packages are available for download at Zenodo^32^. They are included here as Supplementary Software 1 and Supplementary Software 2, respectively, and the associated ReadMe files have been reprinted in Supplementary Notes 6, 7.
