# Supplementary material for: Self-supervised machine learning for live cell imagery segmentation
Source: Commun Biol. 2022 Nov 2;5:1162. doi: 10.1038/s42003-022-04117-x (PMC9630527; doi:10.1038/s42003-022-04117-x)
Supplement: Supplementary file 1 — Supplementary Information [file 42003_2022_4117_MOESM1_ESM.pdf]

Supplementary Information

Self-Supervised Machine Learning for Live Cell Imagery Segmentation

Michael C. Robitaille[1], Jeff M. Byers[1], Joseph A. Christodoulides[1], Marc P. Raphael*[1]

[1] Materials Science and Technology Division, U.S. Naval Research Laboratory, Washington D.C.
* Corresponding author: marc.raphael@nrl.navy.mil

Supplementary Note 1. Static Feature Vectors

The self-supervised algorithm allows for automated training of as many static feature vectors as deemed helpful to robustly segmenting the cells from the background. In this work we utilized the entropyfilt(I,nhood) function in MATLAB to calculate the entropy of image I from a surrounding neighborhood of pixels, nhood, which was fixed to a 7 x 7 matrix based on results from systematic studies of window sizes. In a similar manner, we utilized the imgradient(I,method) function to assign a gradient to a given pixel based on 5 x 5 matrix neighborhood calculated with the 'intermediate' gradient operator method. An example of these feature vector histograms from Dictyostelium time course imagery are shown in Fig S1. Whether a pixel was labeled 'cell' or 'background' was determined by OF. The feature vectors are incorporated into a Naïve Bayesian classifier model in which the prior probabilities are the respective relative frequencies of the 'cell' and 'background' classes.

Measured run times per frame on a laptop computer running Windows 10 for a variety of frame sizes:

512 x 672, 8 bit: 5 sec

1216 x 1920, 8 bit: 7 sec

2050 x 2050, 8 bit: 45 sec

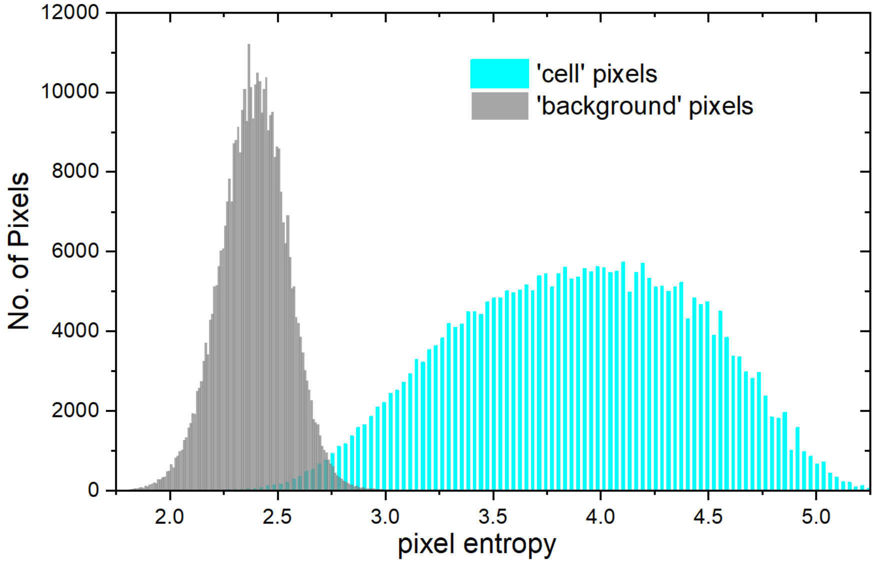

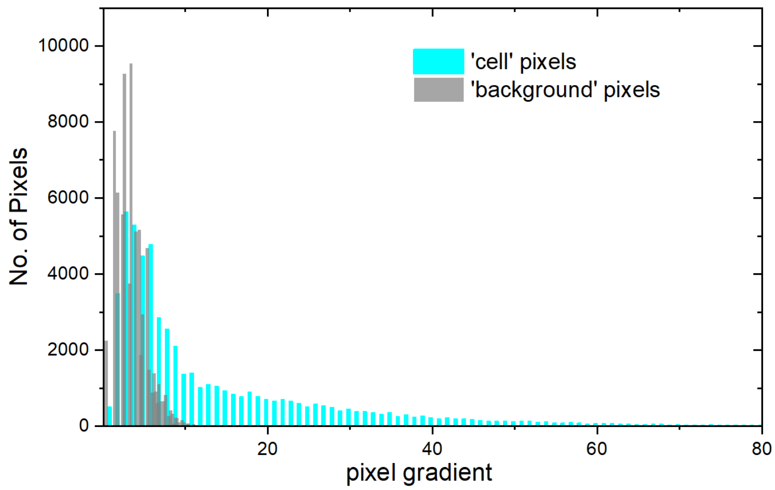

Fig S1. (top) Associated entropy feature vector histograms and (bottom) gradient feature vector histograms of the 'cell' and 'background' training pixels used for model training from transmitted light Dictyostelium images (10X objective).

Supplementary Note 2. Microscopy Details

Main Text Figure	Cell Type	Optical Modality	Objective Magnification	Objective Numerical Aperture	Camera	Wait Time [t-1, t] (seconds)
**Figure 2**	MDA-MB-231 (human breast adenocarcinoma)	DIC	20X	0.8 (air)	Zeiss Axiocam 702 CMOS	300
**Figure 3a**	Hs27 (human foreskin, fibroblast)	Phase	10X	0.45 (air)	Hamamatsu ORCA R2 CCD	1200
**Figure 3b**	Dictyostelium Discoideum (amoeboid)	TL	10X	0.3 (air)	Zeiss Axiocam 702 CMOS	60
**Figure 3c**	MDA-MB-231 (human breast adenocarcinoma)	Phase	10X	0.3 (air)	Zeiss Axiocam 702 CMOS	600
**Figure 3d**	Hs27 (human foreskin, fibroblast)	IRM	40X	1.4 (oil)	Hamamatsu ORCA R2 CCD	600
**Figure 3e**	MDA-MB-231 (human breast adenocarcinoma)	DIC	20X	0.8 (air)	Zeiss Axiocam 702 CMOS	120
**Figure 3f**	A549 (human lung adenocarcinoma)	Fluorescence (LifeAct)	100X	1.46 (oil)	iXon Ultra EMCCD	10

Table S1 Cell and optical details for imagery in the main text. DIC: Differential Interference Contrast; Phase: Phase Contrast; TL: Transmitted Light illumination; IRM: Interference Reflection Microscopy.

Supplementary Note 3. Fitting Surfaces To Images To Find The Displacement Field

We can think of a grayscale image, I, as a cloud of points in three dimensions where the intensity of the pixel is the height, $z = I(i,j)$, above the corresponding locations (i,j) in the image plane with coordinates x and y (Fig S2). A natural idea is to fit a smooth function, $F(x,y)$, to the discrete samples represented by the image, $I(i,j)$. Unlike the image itself, the function is defined at every location (x,y) in the plane of the image not just the locations of the pixels (i,j). Also, the function has a continuum of intensity values not just at the discrete values of the image format (e.g, the integers from 0-255 for 8-bit images). Now, consider fitting a function to each of a pair of consecutive images in time, I_1 and I_2. The central premise of optical flow is that the corresponding functions, F_1 and F_2, can be related to each other by a spatial shifting of pixel values, called a displacement field, $\boldsymbol{d}(x,y)$: $F_2 = F_1(\boldsymbol{x} - \boldsymbol{d})$ where $\boldsymbol{x} = (x,y)$.

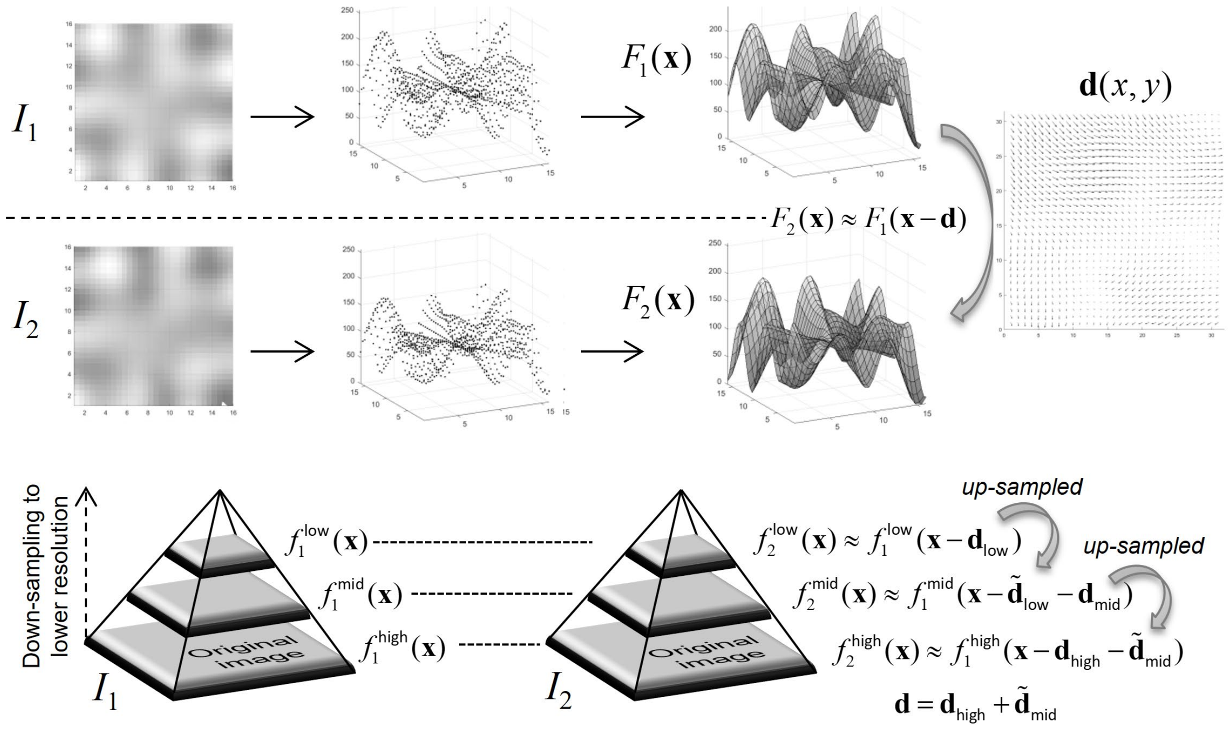

Fig S2. An example using a three-level image pyramid to compute the displacement field, $\boldsymbol{d}(x,y)$. The original image pair, I_1 and I_2, is progressively down-sampled to create lower-resolution versions. Image surface functions, f, are fit to local patches in the images at each level of the pyramid. Beginning with the lowest resolution functions, f_1^{low}, and f_2^{low}, a displacement field, $\mathbf{d}_{\mathrm{low}}$, is calculated and, then, up-sampled, $\tilde{\mathbf{d}}_{\mathrm{low}}$, to the resolution of the middle layer images in the pyramid. This process is repeated for the middle layer to provide an initial displacement field, $\tilde{\mathbf{d}}_{\mathrm{mid}}$, for the determining the final displacement field, **d**, between the original images.

Optical flow methods differ on how this displacement field is computed. Global methods (e.g., Horn-Schunck algorithm[1]) solve for the displacement field across the entire image simultaneously and iterate this process to achieve a prescribed level of smoothness. While sensitive to noise, this approach can

create a dense displacement field even in the interior of constant brightness regions. Local methods (e.g., Lucas-Kanade algorithm[2]) solve for the displacement field in local neighborhoods of pixels or patches using linear regression, typically. This approach is more robust to noise but requires very small differences between the local patches being compared between images and is effective at high image acquisition rates compared to motion in the images. Another class of optical flow methods (e.g. Farneback algorithm[3]) operate between the global and local forms via a multi-resolution approach that iteratively determines the displacement field between a series of lower resolution versions of the original images.

The multi-resolution approach combines the robustness to noise and global image shifts while being able to track the deformation of cells occurring at varying length scales and microscope magnifications. We have chosen this optical flow method for the automatic semantic segmentation module of our self-supervised learning approach. The method we use corresponds to fitting a 2nd-order polynomial in x and y to a local patch of the image and then repeating this process across the entire image to approximate the surface functions. As already described for local methods, this patch-wise reshaping of the image surface f_1 into f_2, at a particular resolution is more effective if the shifts are not large compared to the size of neighborhood used to fit the polynomials in each image.

Optical flow assumptions may or may not be met for fluorescence time-lapse imagery applications in which extended time intervals are sometimes employed to avoid phototoxicity or photobleaching.[4,5] For this reason, it was important that our technique be co-validated with tag free techniques such as transmitted light and phase contrast which are minimally invasive. Overlays of less frequently accumulated fluorescence imagery with cells segmented using a tag-free imaging channel is then straightforward.

Supplementary Note 4. Segmented Cell Area as a function of Smoothing Disk Size

Hole filling was accomplished with the imclose(I,SE) function in Matlab in which I is the binary input image with ‘cell’ pixel values 1 and ‘background’ pixel values 0, and SE is structural element whose size and shape determines the extent of the blurring. We used a disk SE of radius *r* and by plotting the average segmented cell area in the field of view as a function of r we determined a range in which the area output remained relatively constant across all cell types and optical modalities. Fig S3a shows the mean segmented area of Hs27 cells (phase contrast, 10X magnification) versus r. From $1 < r < 11$ the segmented area is stabilized, while above $r = 11$ discrete steps in area are observed as a result of cells being grouped together as shown in the cell segmentation images of Fig S3b for $r = 5$ pixels versus Fig S3c for $r = 20$ pixels. The $r = 5$ pixel value was found to reliably hole fill for all cells and optical modalities in this study.

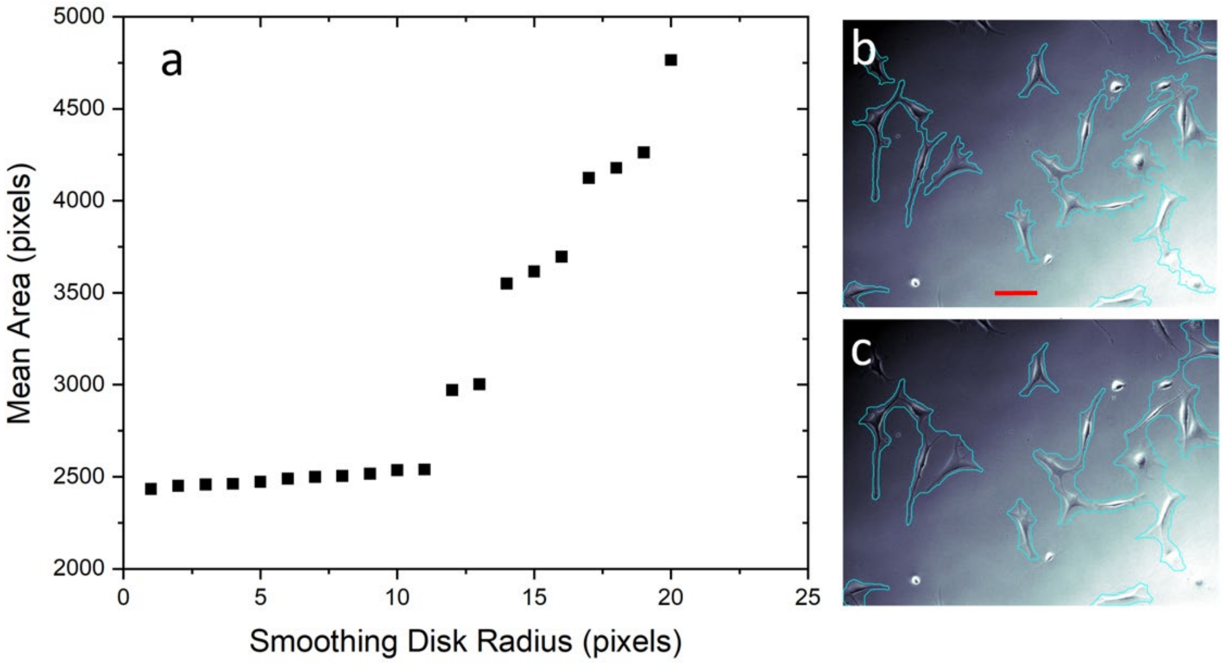

Fig S3. a. Mean area of the segmented cells in the field of view as a function of the smoothing disk radius. Segmented images of cells with **b.** *r = 5* pixels and **c.** *r = 20* pixels. Scale bar 50 µm.

Supplementary Note 5. Method Evaluation

To compare the performance of our self-supervised approach with contemporary methods, we utilized the recent CellPose[6] generalist model on the same image set summarized in the main text Fig 3 and tabulated in Table S1. CellPose offers supervised pixel classification, however CellPose relies largely upon a pre-established dataset of highly varied images of cells containing over 70,000 segmented objects, with the option of users to submit their own labeled data to be incorporated into the training library at a later point in time. In this regards, CellPose is an offline machine learning method, with the supervision burden shifted from the users onto the developers. As such, we opted to rely upon the heavily trained library for CellPose for segmentation of the validation data set used in our study. Thus, no manual annotation was conducted, instead the large pre-trained library was applied to the data set for comparison. All segmentation was done using the cytoplasm model, and the size was initially automatically determined by the calibrate option, unless manually entered diameters were found to provide better segmentation. The figures below show the ground truth (green-solid lines), SSL (cyan-large dashes), and CellPose (red-small dashes) outlines overlaid on the final image of the data set.

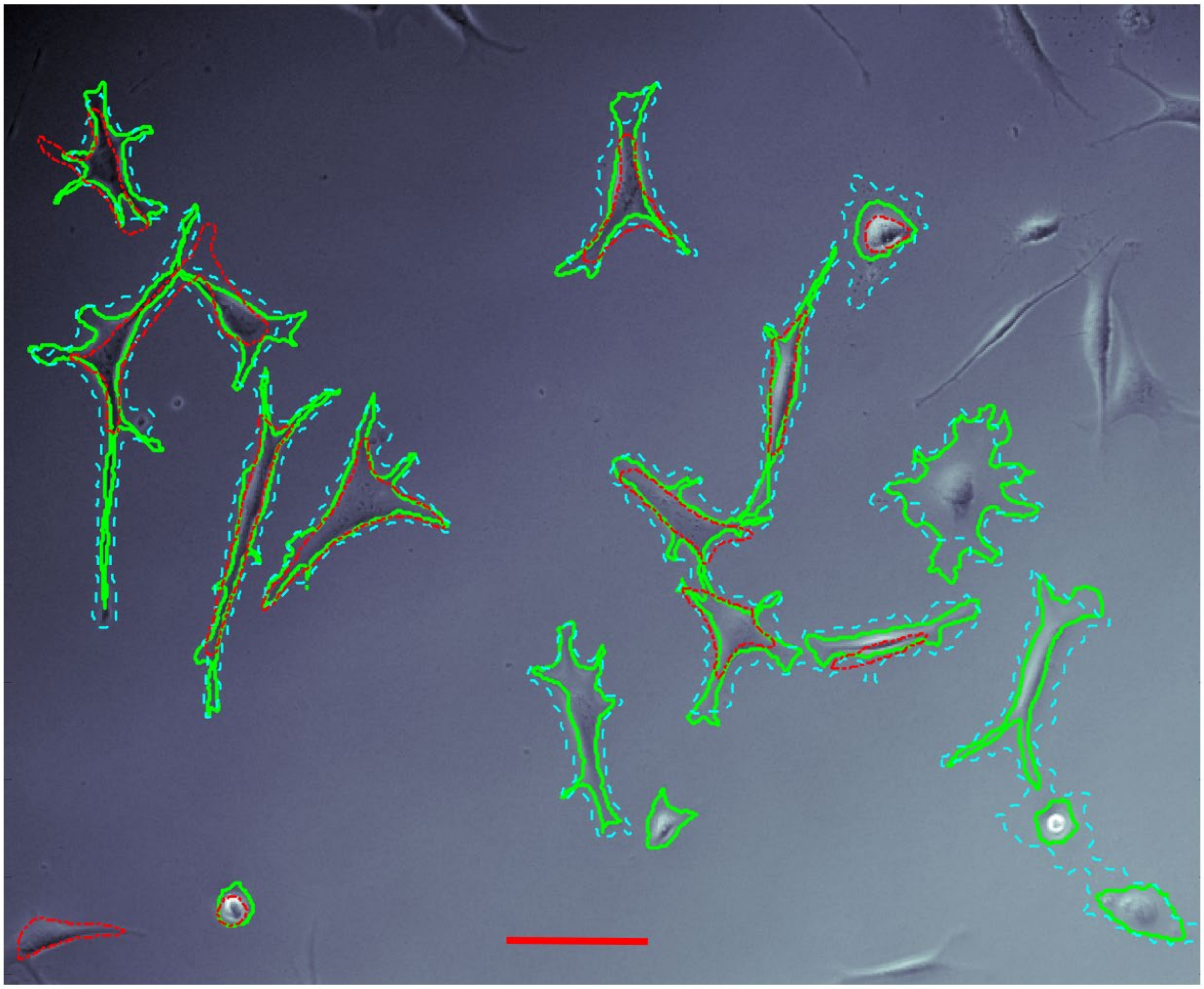

Fig S4. Hs27 10x phase segmentation overlays for comparison. Scale bar 50 µm.

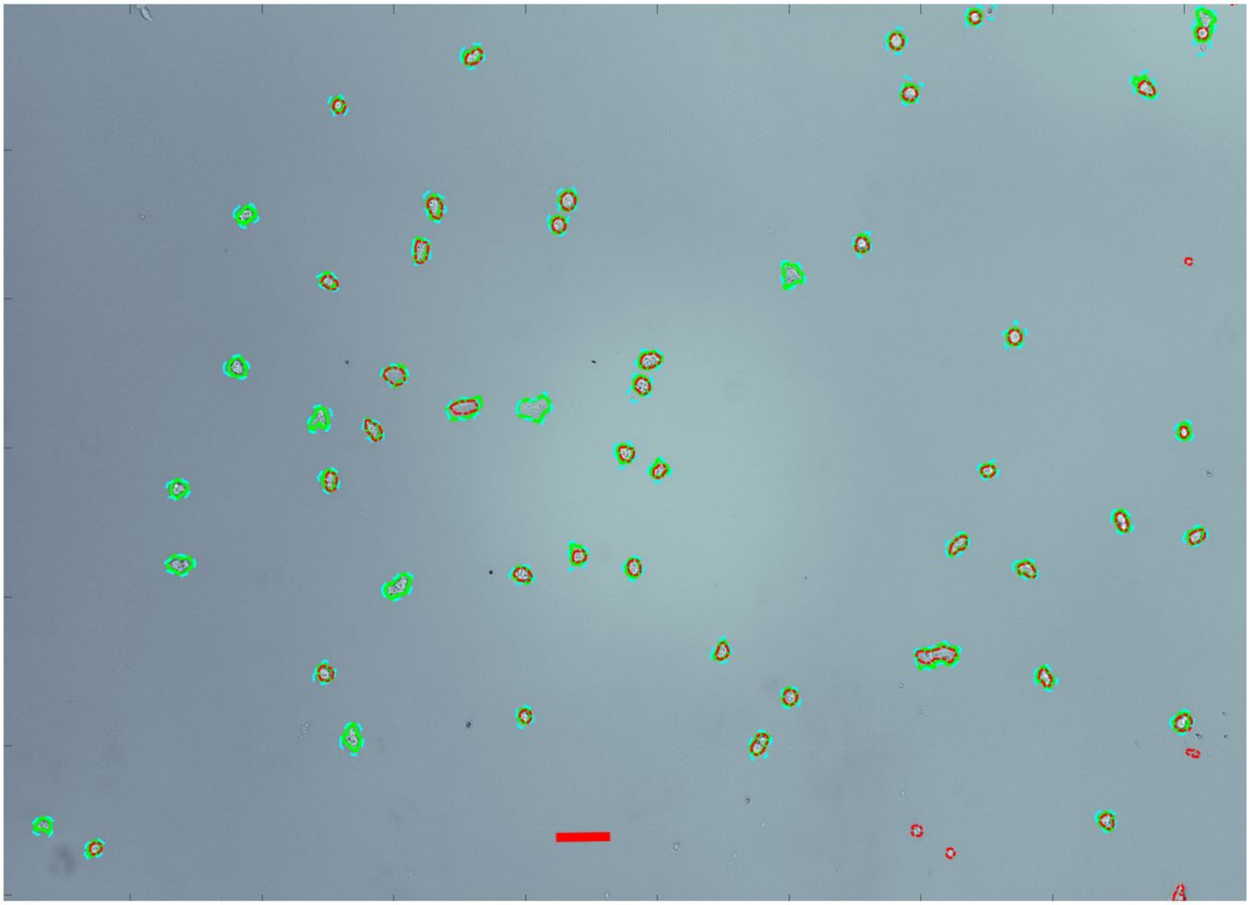

Fig S5. Dicty 10x TL segmentation overlays for comparison. Scale bar 50 µm.

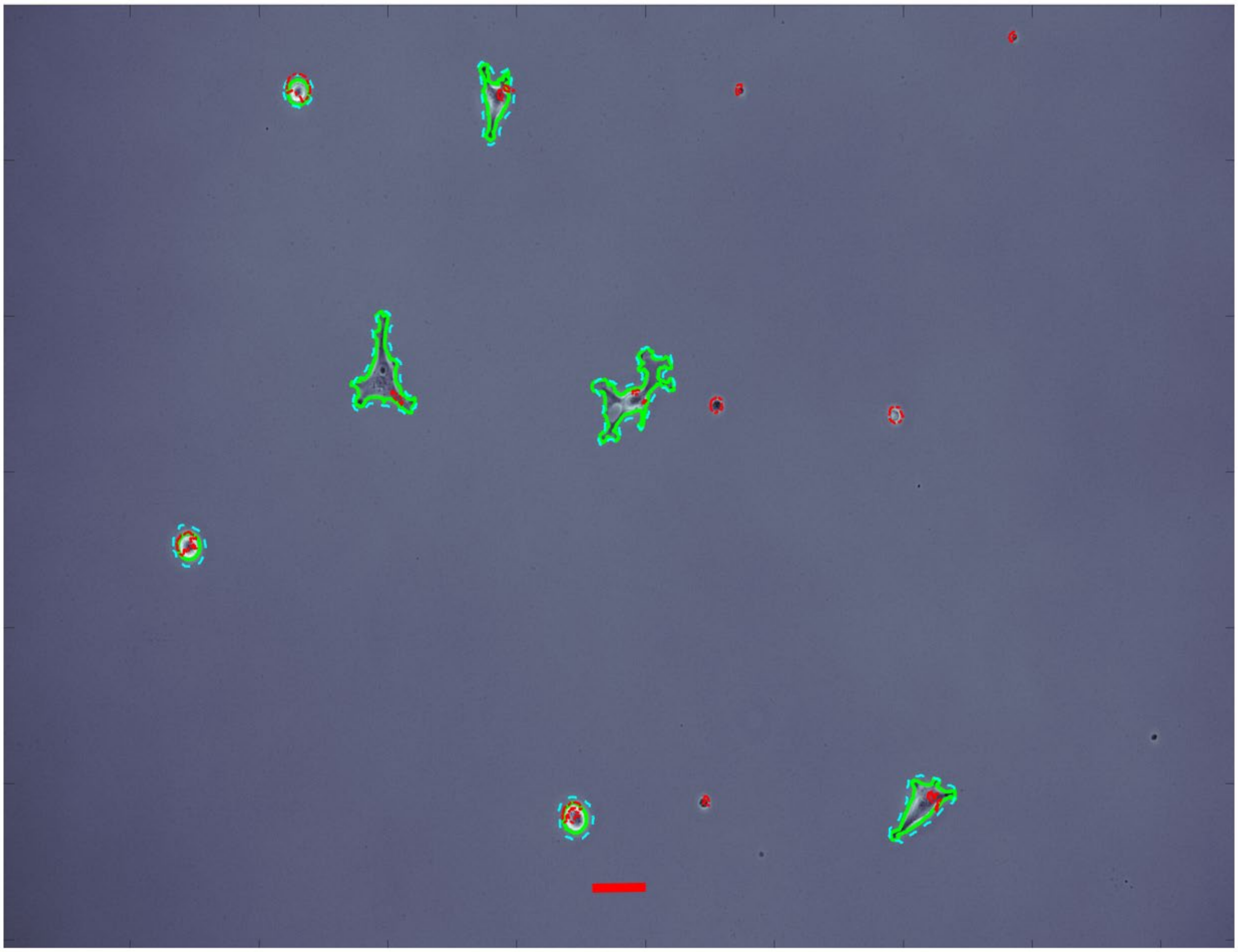

Fig S6. MDA-MB-231 10x phase segmentation overlays for comparison. Scale bar 50 µm.

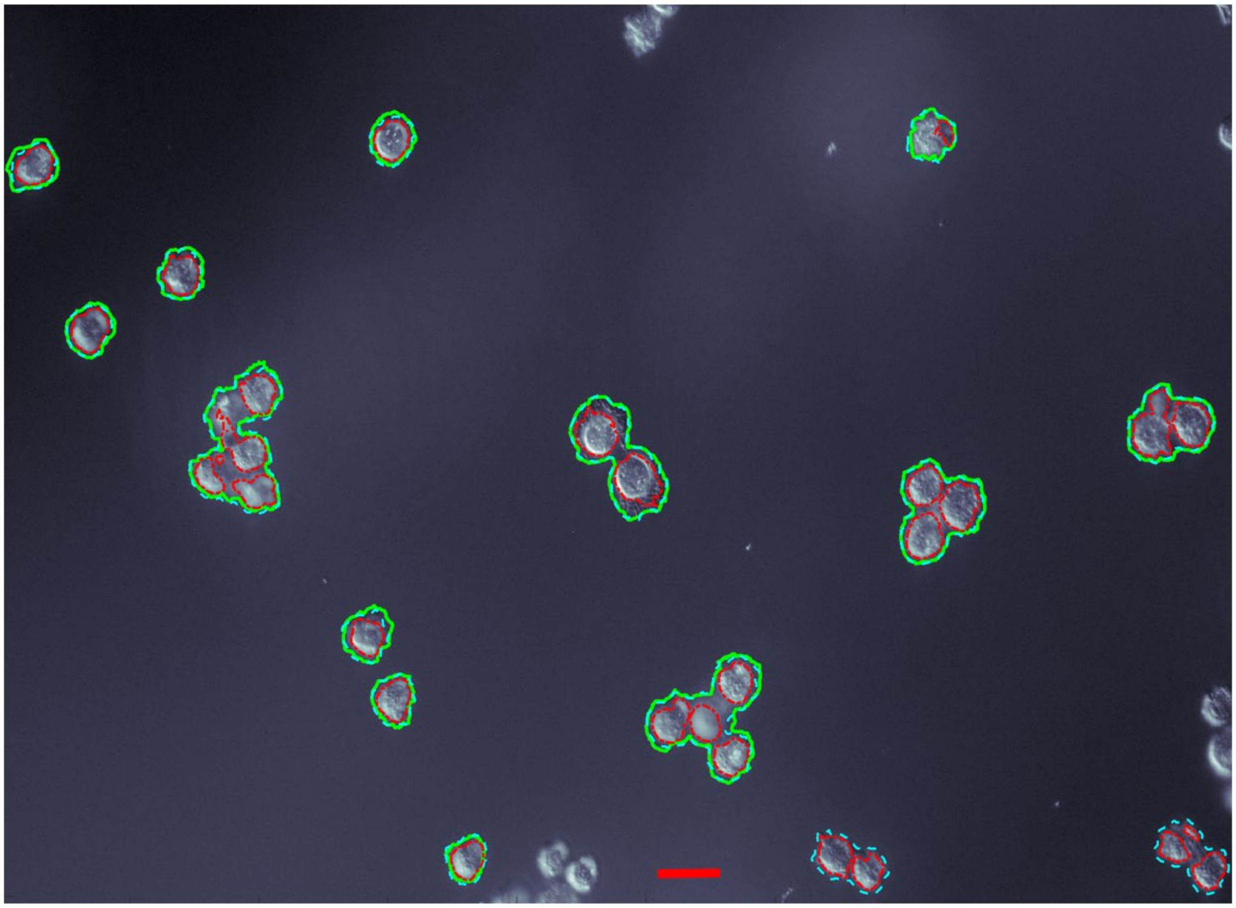

Fig S7. MDA-MB-231 20x DIC segmentation overlays for comparison. Scale bar 25 µm.

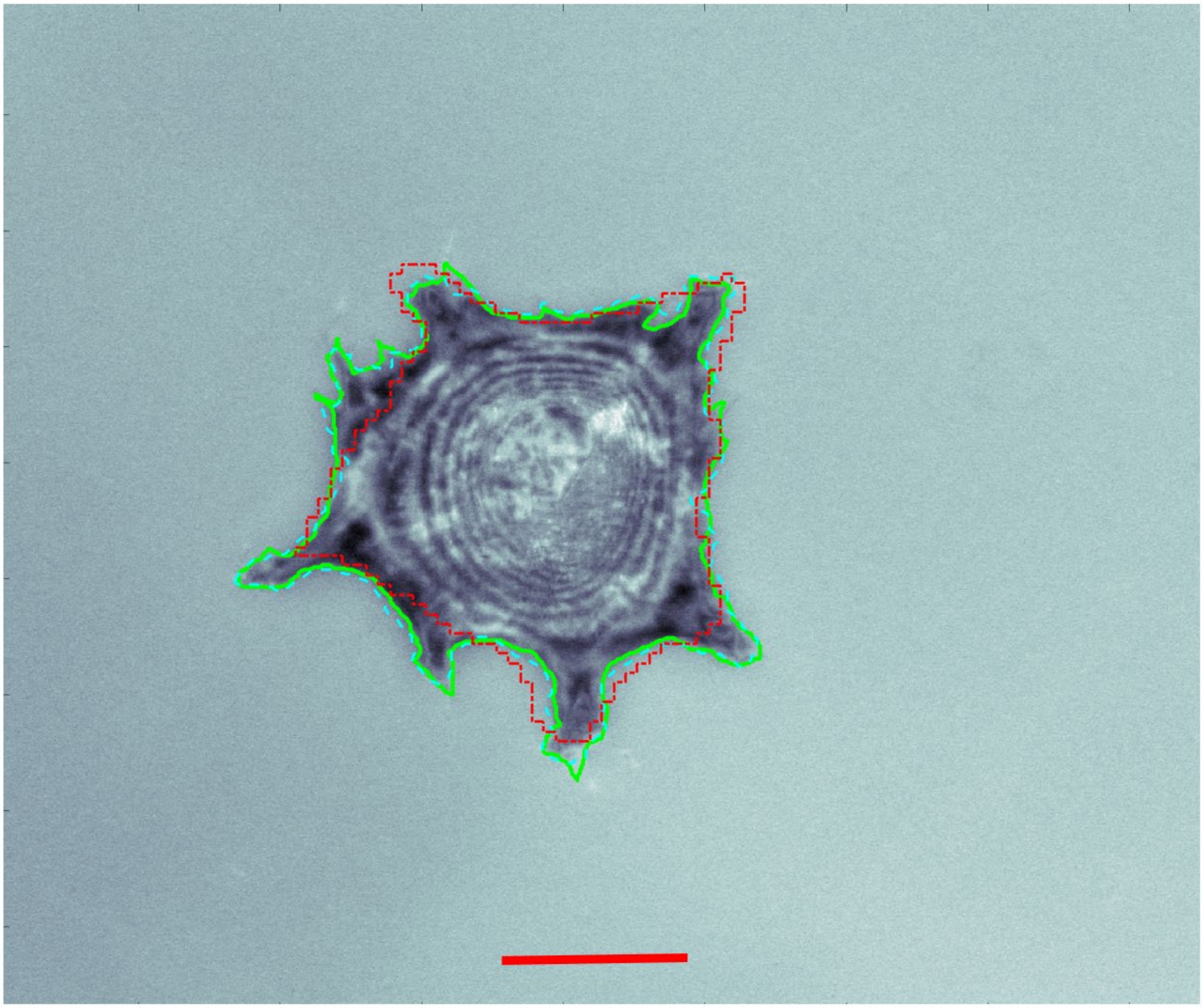

Fig S8. Hs27 40x IRM segmentation overlays for comparison. Scale bar 25 µm.

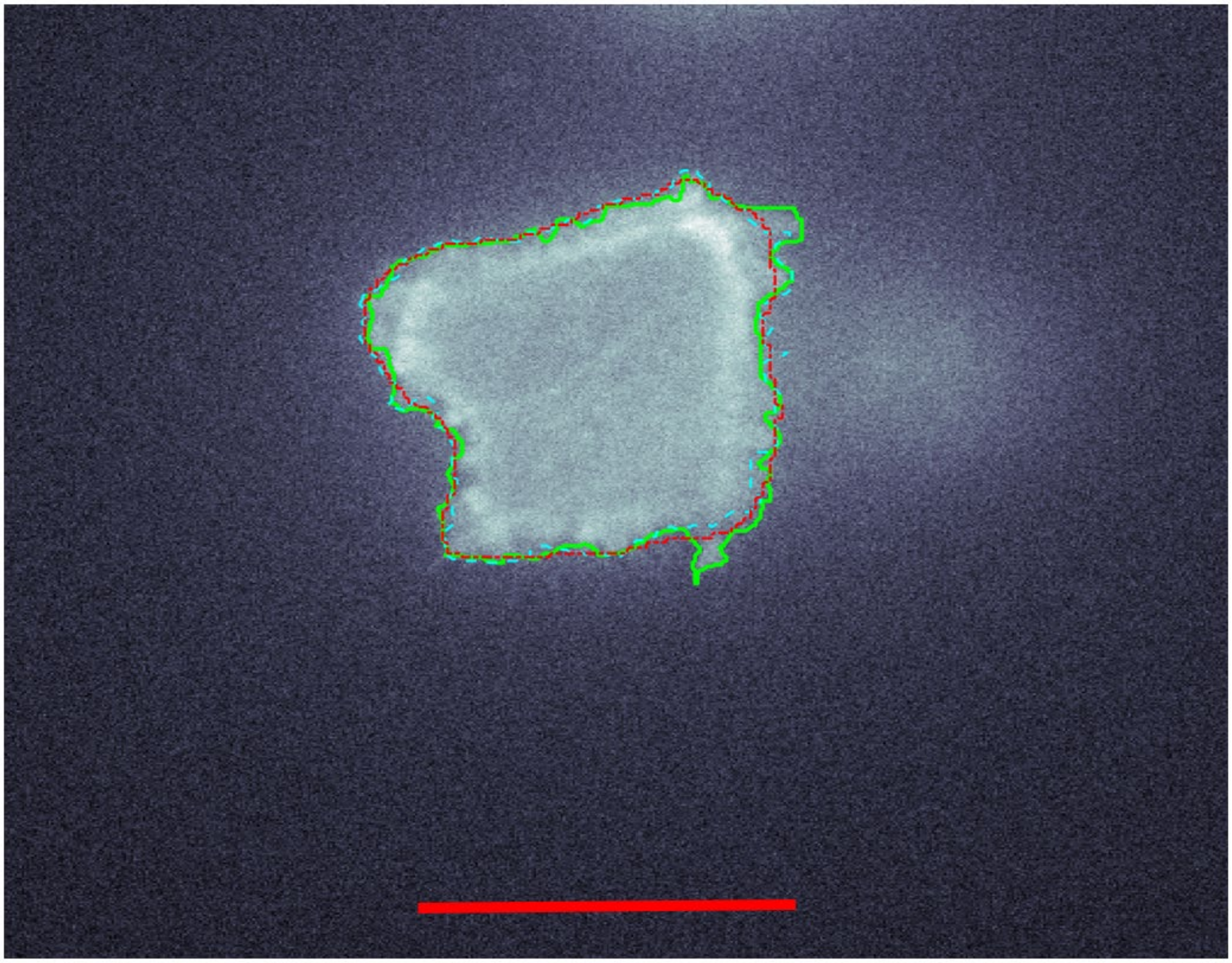

Fig S9. A549 100x fluorescence segmentation overlays for comparison. Scale bar 10 µm.

Supplementary Note 6. GUI ReadMe File

Self-supervised machine learning code for segmenting live cell imagery.

The included stand-alone graphical user interfaces (GUIs) are designed to be used with time-resolved live cell microscopy images (tiffs) for the automated segmentation of cells from background. There is a dedicated stand-alone GUI for Windows, Mac and Linux operating systems (OS) :

SSL_Win: Tested on Windows 10 OS
SSL_Mac: Tested on macOS Big Sur Version 11.0.1
SSL_Linux: Tested on Linux Ubuntu 20.04.4 LTS

The GUIs were generated using the Matlab Application Compiler and require Matlab Runtime. A free download of Matlab Runtime can be found here:

https://www.mathworks.com/products/compiler/matlab-runtime.html

Download the 64-bit version zip file associated with your OS of choice using the version indicated by the red boxes below. Extract the zipped files, double click 'setup' and follow the installation instructions.

Release (MATLAB Runtime Version#)	Windows	Linux	Mac
R2022a (9.12)	64-bit	64-bit	Intel 64-bit
R2021b (9.11)	64-bit	64-bit	Intel 64-bit
R2021a (9.10)	64-bit	64-bit	Intel 64-bit

The principle of self-supervised machine learning is that you simply load your images and hit 'Run' - no parameter tuning needed, no training imagery required.

Run from start to finish, the code uses consecutive pairs of images to generate unsupervised training data of 'cells' and 'background' via dynamic feature vectors based on optical flow. These self-labeled pixels are then used to generate static feature vectors (*e.g.* entropy, gradient), which in turn are used to train a classifier model. The training data is updated every image in order to automatically adapt to temporal changes in cell morphologies or background illumination.

This demo code allows the user to reproduce figures from the main manuscript or work with their own images. As an additional example, a short time series of 15 phase contrast images has been included (see 'Time_Series_Sample_Imagery' folder).

To use this code:

1. Unzipped SSL_GUI_Demo_Package
2. Run either SSL_Win, SSL_Mac or SSL_Linux based on your OS
3. Give the GUI several seconds to open (it may take some time for Matlab Runtime to start up in the background)

4. You can select the included imagery from Figure 3 in the manuscript or select at least two images from the “Time_Series_Sample_Imagery” images.
5. You can also add your own data (at least two images) to the \UserData\data folder
6. If the “Select Data from Paper” option gives an error, use the “Select User Data” option and select your tiff files (at least two) from the desired Data folder.
7. Either way, the code will segment the image and create an *.avi movie one directory level above.
8. Note that a Matlab binary mask (*_mask.mat) file of the segmented cells will also be created for future use if desired.

Supplementary Note 7. Matlab Code ReadMe File

Self supervised machine learning code for segmenting live cell imagery.

This Matlab code is designed to be used with time-resolved live cell microscopy images (tiffs) for the automated segmentation of cells from background. This code was tested on Matlab v2020a, v2021a and v2022a using commercially available laptop computers running the Windows 10 operating system. The code requires the following additional toolboxes to be installed:

Computer Vision Toolbox
Image Processing Toolbox
Statistics and Machine Learning Toolbox

Matlab and the associated toolboxes are available for free 30 day trials if not currently licensed by your institution.

The principle of self-supervised machine learning is that you simply load your images and hit 'Run' - no parameter tuning needed, no training imagery required.

Run from start to finish, the code uses consecutive pairs of images to generate unsupervised training data of 'cells' and 'background' via dynamic feature vectors based on optical flow. These self-labeled pixels are then used to generate static feature vectors (*e.g.* entropy, gradient), which in turn are used to train a classifier model. The training data is updated every image in order to automatically adapt to temporal changes in cell morphologies or background illumination.

This demo code allows the user to reproduce figures from the main manuscript or work with their own images. As an additional example, a short time series of 15 phase contrast images has been included (see 'Time_Series_Sample_Imagery' folder).

To use this code:

1. Place the unzipped SSL_Matlab_Demo_Package folder in your Matlab working directory
2. Open SSL_Demo_NC.m
3. Run the code
4. When prompted in the command window, answer whether or not you want to reproduce a figure from the paper.
5. If yes, you will be prompted for the Fig 3 letter (a, b, c…)
6. If no, you will be prompted to first place your tiff imagery (or the included time series sample imagery) into the SSL_Demo_Package\UserData\data folder
7. Either way, the code will segment the imagery, place a binary mask of the segmented cells in the associated 'mask' folder and create an *.avi movie one directory level above.

SUPPLEMENTARY REFERENCES

1 Horn, B. K. & Schunck, B. G. Determining optical flow. *Artificial intelligence* **17**, 185-203 (1981).

2 Lucas, B. D. & Kanade, T. An iterative image registration technique with an application to stereo vision. (1981).
3 Farnebäck, G. in *Scandinavian conference on Image analysis.* 363-370 (Springer).
4 Schroeder, T. Long-term single-cell imaging of mammalian stem cells. *Nature Methods* **8**, S30-S35, doi:10.1038/nmeth.1577 (2011).
5 Skylaki, S., Hilsenbeck, O. & Schroeder, T. Challenges in long-term imaging and quantification of single-cell dynamics. *Nature Biotechnology* **34**, 1137-1144, doi:10.1038/nbt.3713 (2016).
6 Stringer, C., Wang, T., Michaelos, M. & Pachitariu, M. Cellpose: a generalist algorithm for cellular segmentation. *Nature Methods* **18**, 100-106 (2021).
