# Supplementary material for: Self-supervised machine learning for live cell imagery segmentation
Source: Commun Biol. 2022 Nov 2;5:1162. doi: 10.1038/s42003-022-04117-x (PMC9630527; doi:10.1038/s42003-022-04117-x)
Supplement: Supplementary file 2 — Description of Additional Supplementary Files [file 42003_2022_4117_MOESM2_ESM.pdf]

Description of Additional Supplementary Files

File name: Supplementary Data
Description: TIFF imagery evaluated in Figure 3.

File name: Supplementary Software 1
Description: Stand alone graphical user interface software. Includes instantiations for Windows, Mac and Linux operating systems.

File name: Supplementary Software 2
Description: Matlab source code used to generate the results with options for the user to run their own imagery.
