# Supplementary figures and images for: Self-supervised machine learning for live cell imagery segmentation
Source: Commun Biol. 2022 Nov 2;5:1162. doi: 10.1038/s42003-022-04117-x (PMC9630527; doi:10.1038/s42003-022-04117-x)

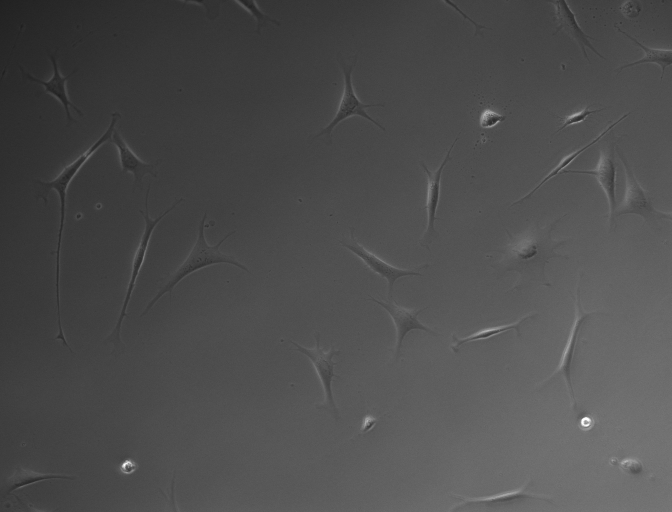

Supplement: Supplementary file 3 — Supplementary Data [file 42003_2022_4117_MOESM3_ESM.zip › Fig3/a/data/Fig3a_t1.tif]

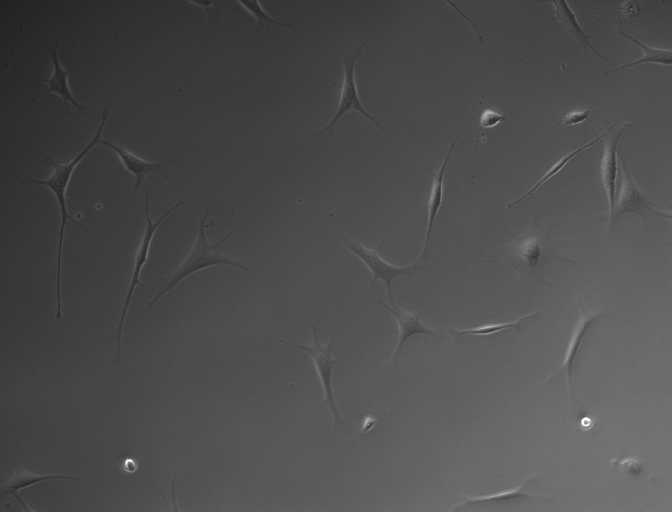

Supplement: Supplementary file 3 — Supplementary Data [file 42003_2022_4117_MOESM3_ESM.zip › Fig3/a/data/Fig3a_t2.tif]

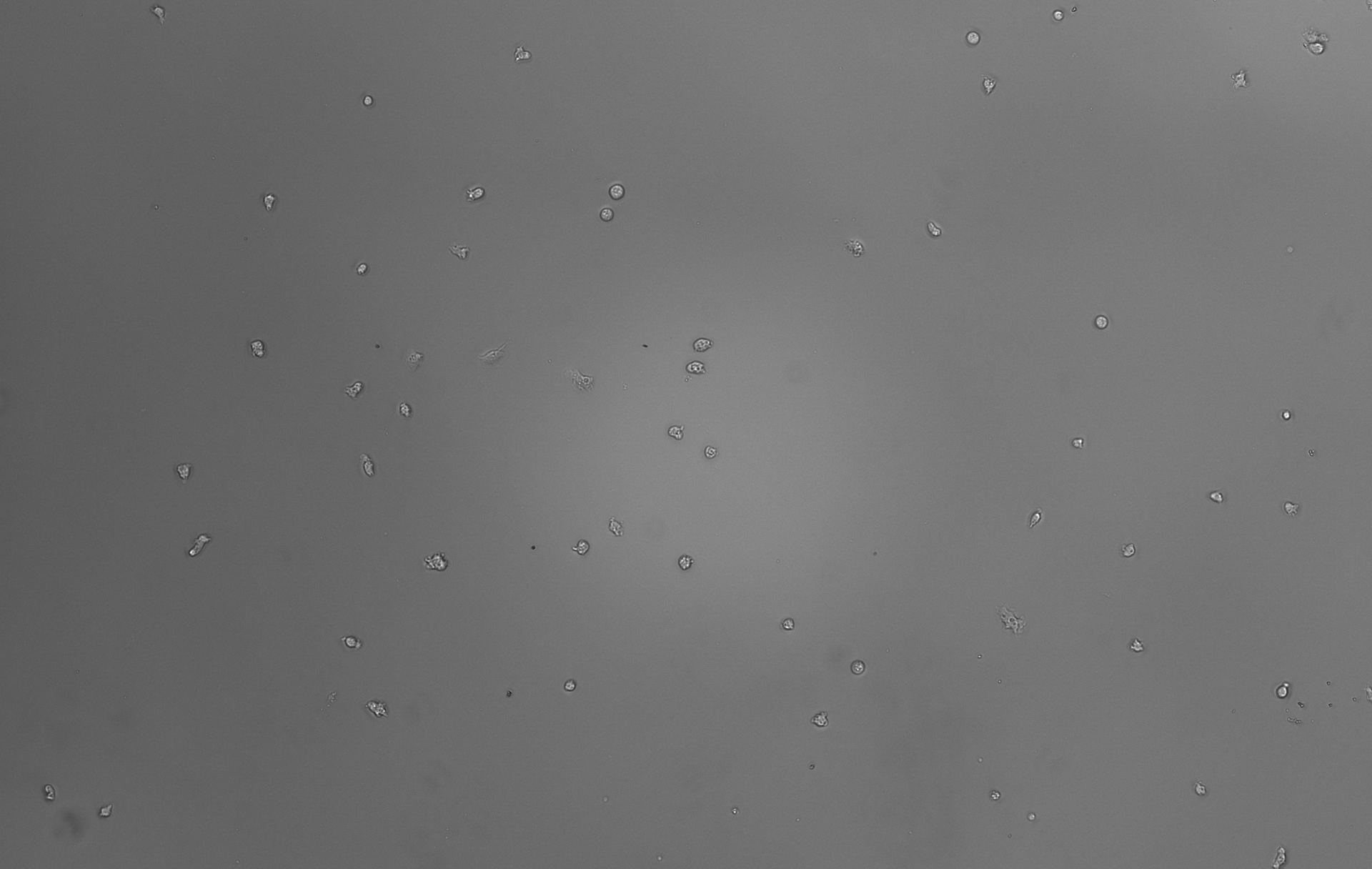

Supplement: Supplementary file 3 — Supplementary Data [file 42003_2022_4117_MOESM3_ESM.zip › Fig3/b/data/Fig3b_t1.tif]

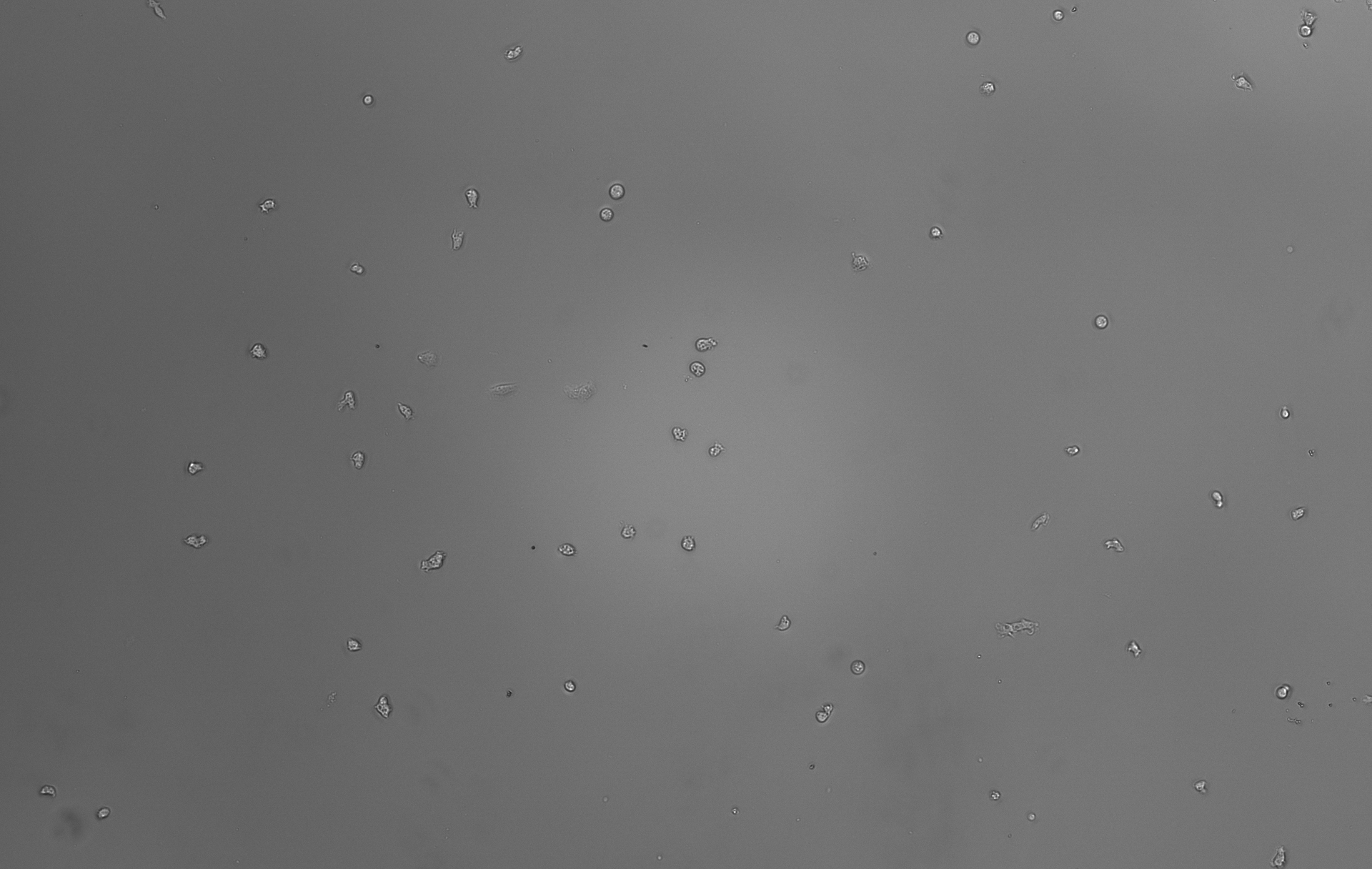

Supplement: Supplementary file 3 — Supplementary Data [file 42003_2022_4117_MOESM3_ESM.zip › Fig3/b/data/Fig3b_t2.tif]

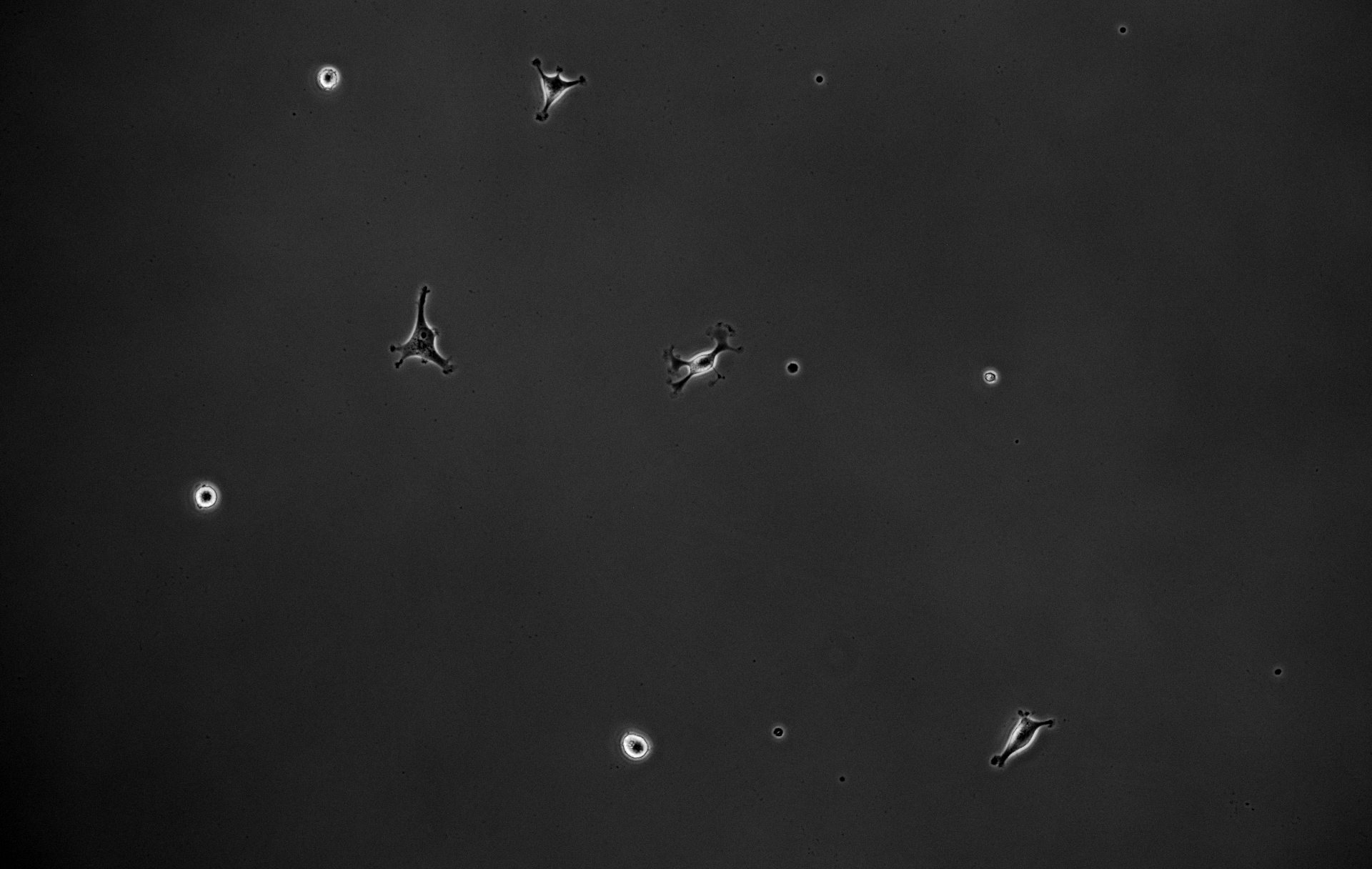

Supplement: Supplementary file 3 — Supplementary Data [file 42003_2022_4117_MOESM3_ESM.zip › Fig3/c/data/Fig3c_t1.tif]

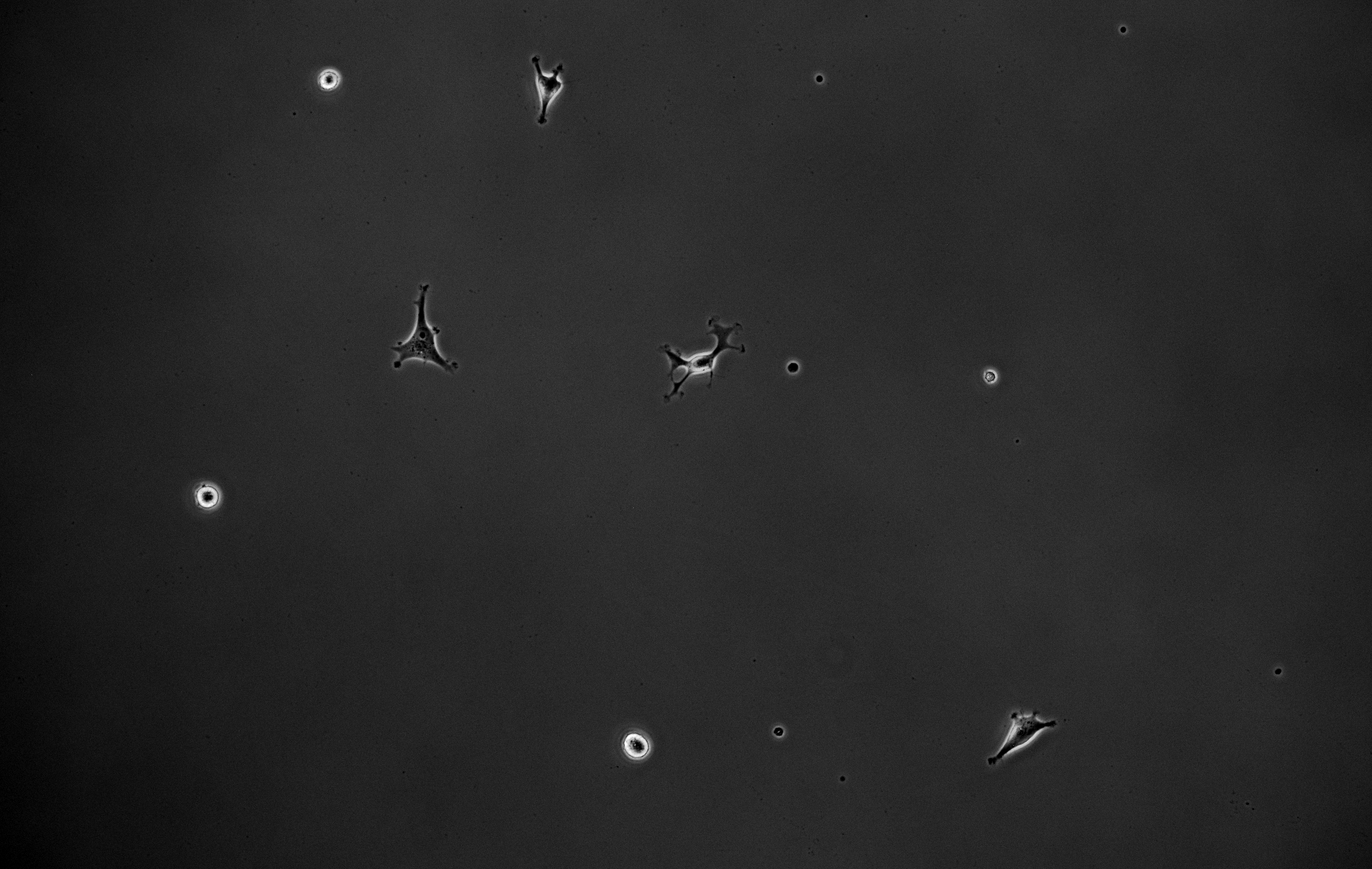

Supplement: Supplementary file 3 — Supplementary Data [file 42003_2022_4117_MOESM3_ESM.zip › Fig3/c/data/Fig3c_t2.tif]

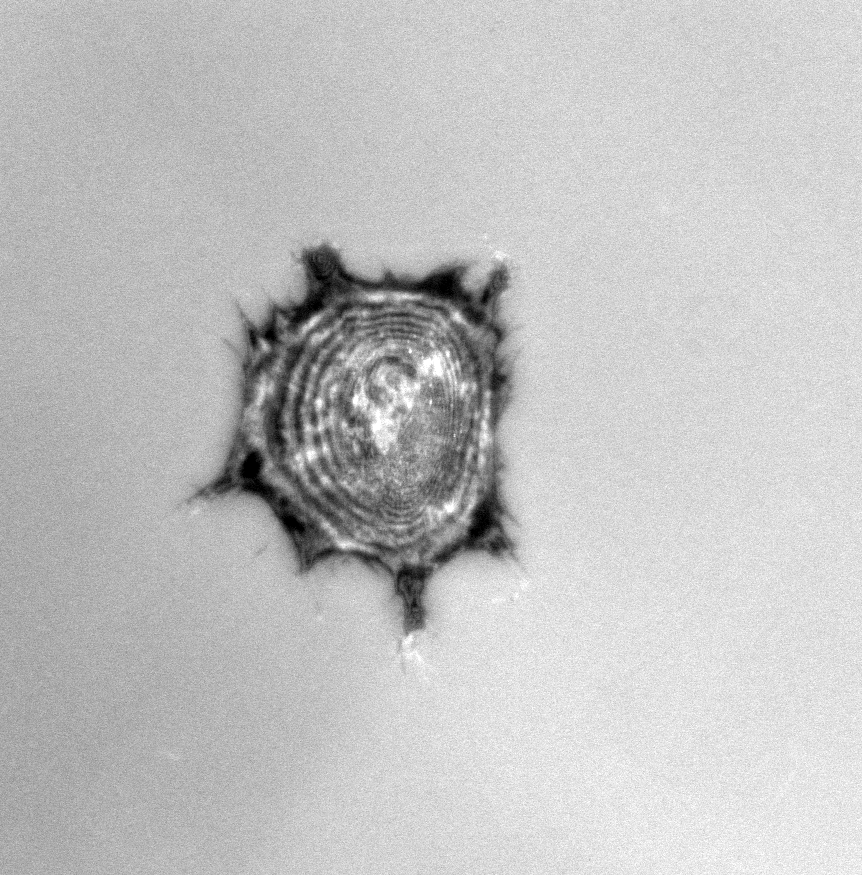

Supplement: Supplementary file 3 — Supplementary Data [file 42003_2022_4117_MOESM3_ESM.zip › Fig3/d/data/Fig3d_t1.tif]

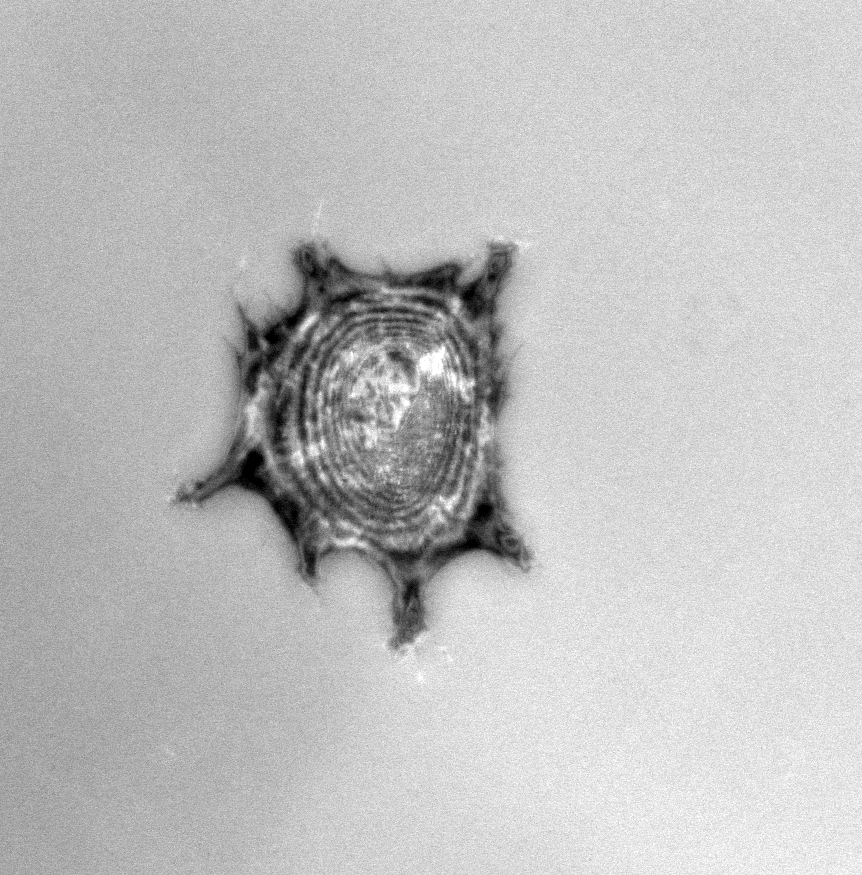

Supplement: Supplementary file 3 — Supplementary Data [file 42003_2022_4117_MOESM3_ESM.zip › Fig3/d/data/Fig3d_t2.tif]

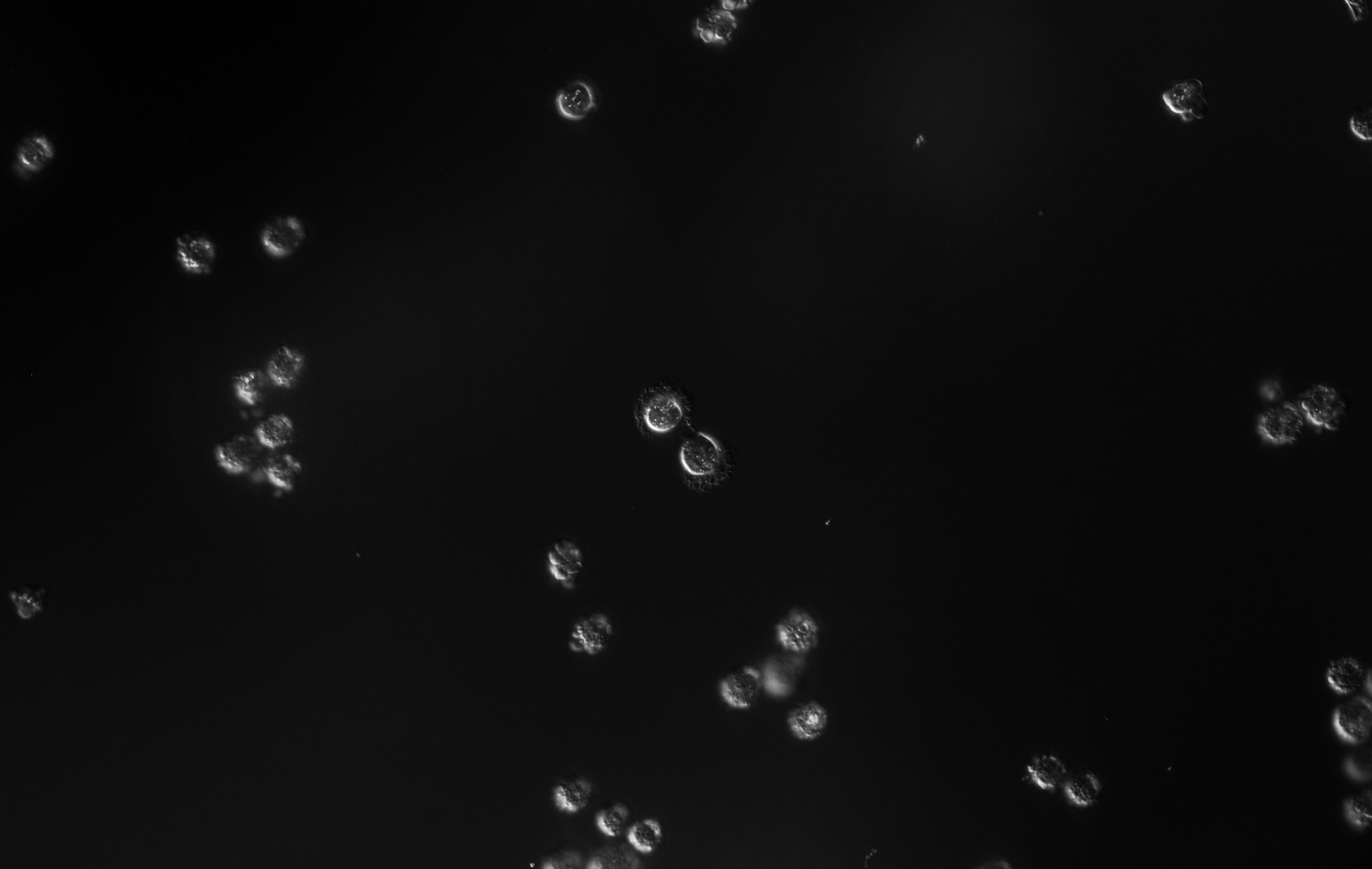

Supplement: Supplementary file 3 — Supplementary Data [file 42003_2022_4117_MOESM3_ESM.zip › Fig3/e/data/Fig3e_t1.tif]

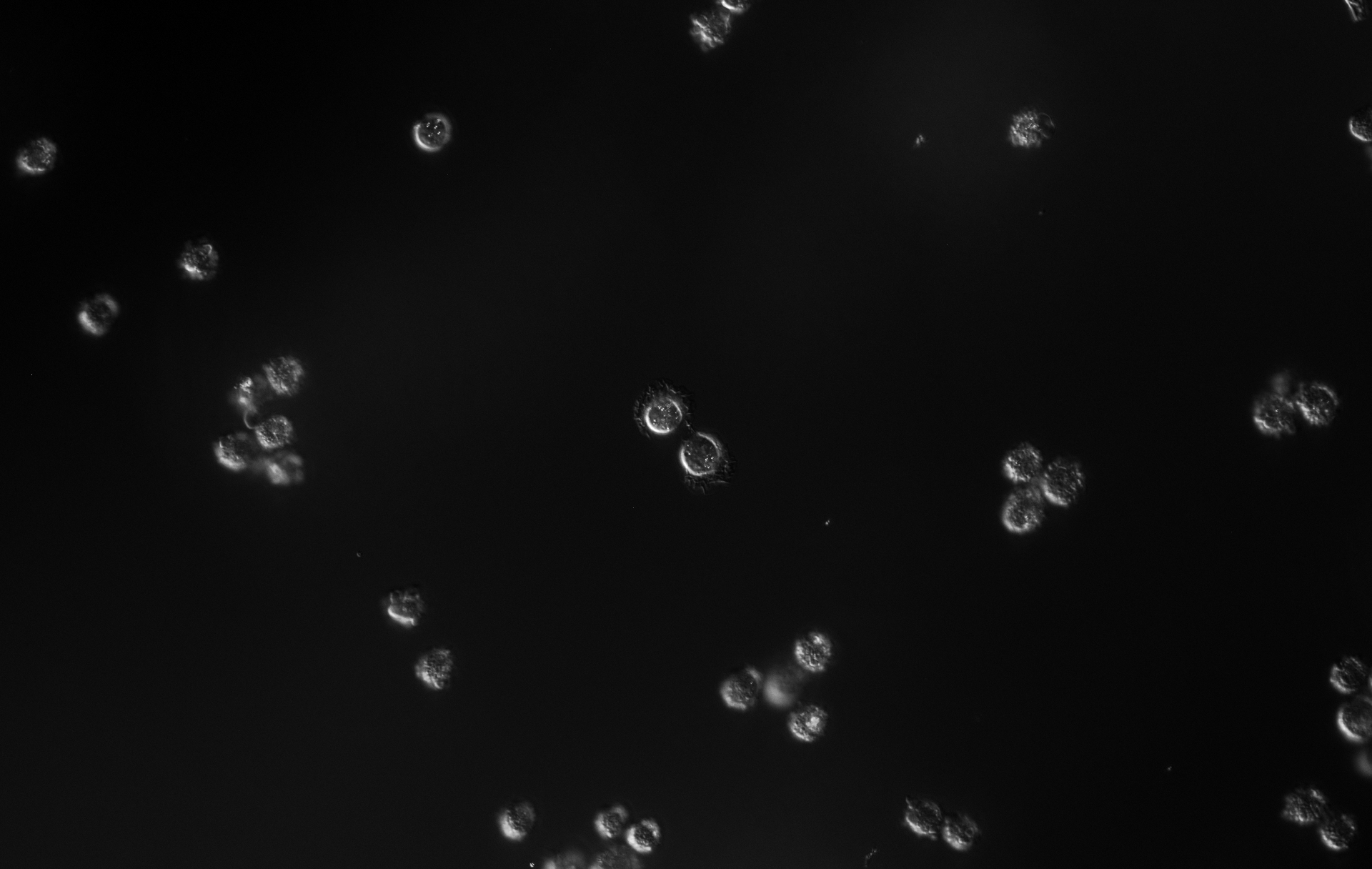

Supplement: Supplementary file 3 — Supplementary Data [file 42003_2022_4117_MOESM3_ESM.zip › Fig3/e/data/Fig3e_t2.tif]

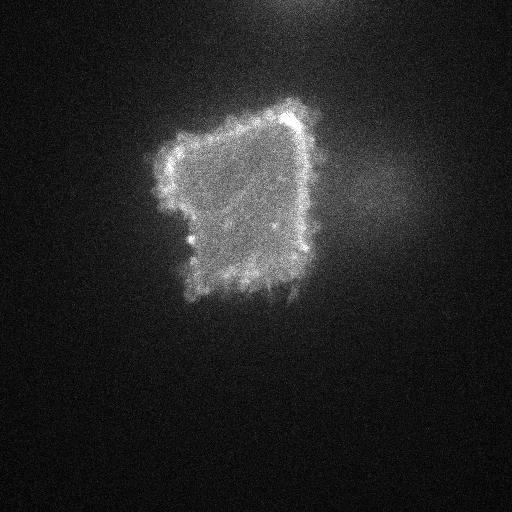

Supplement: Supplementary file 3 — Supplementary Data [file 42003_2022_4117_MOESM3_ESM.zip › Fig3/f/data/Fig3f_t1.tif]

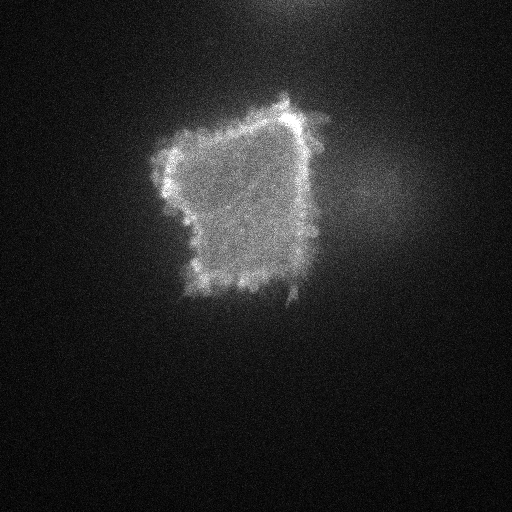

Supplement: Supplementary file 3 — Supplementary Data [file 42003_2022_4117_MOESM3_ESM.zip › Fig3/f/data/Fig3f_t2.tif]

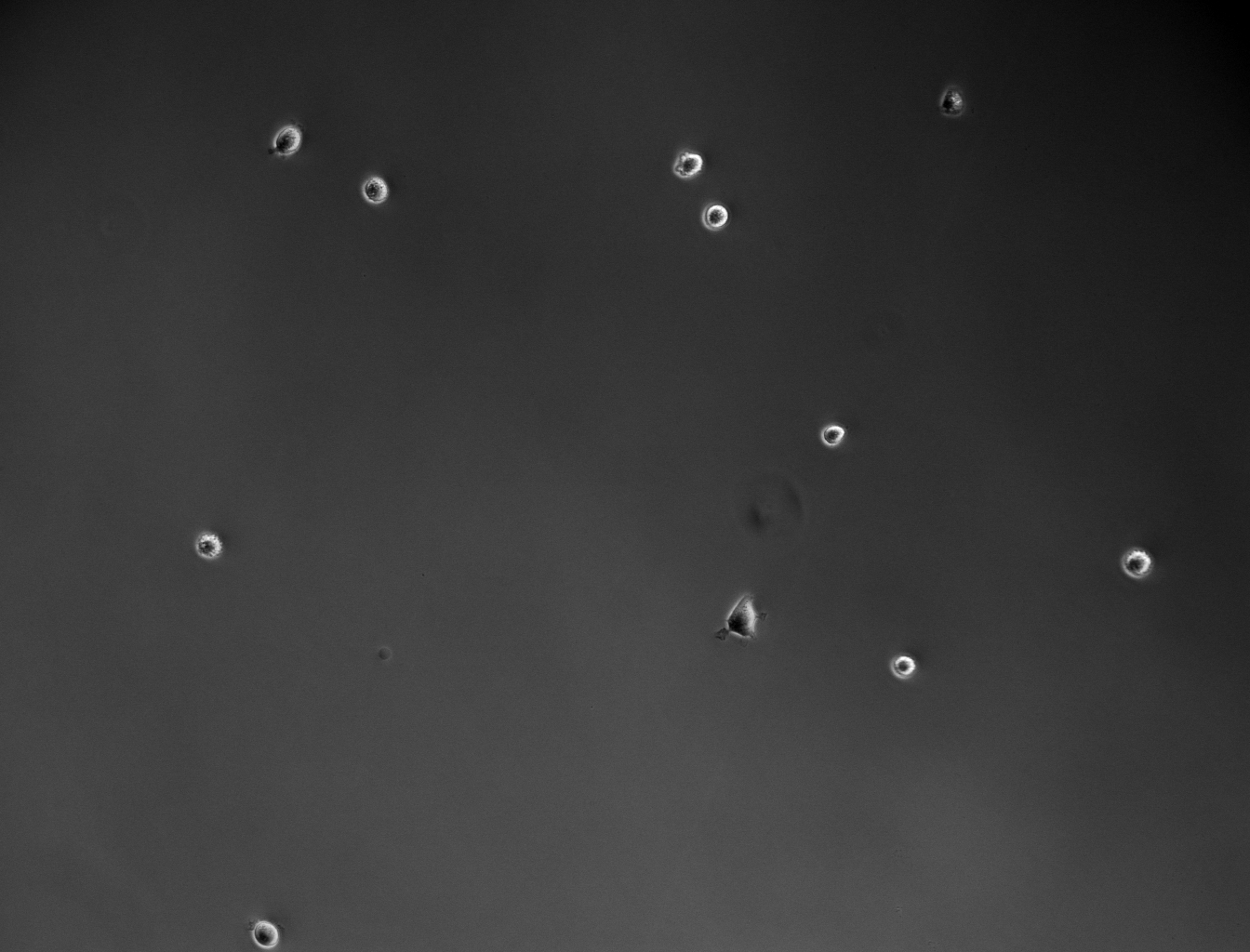

Supplement: Supplementary file 4 — Supplementary Software 1 [file 42003_2022_4117_MOESM4_ESM.zip › SSL_GUI_Demo_Package/Time_Series_Sample_Imagery/MDA_MB_231_Phase_10X_t001.tif]

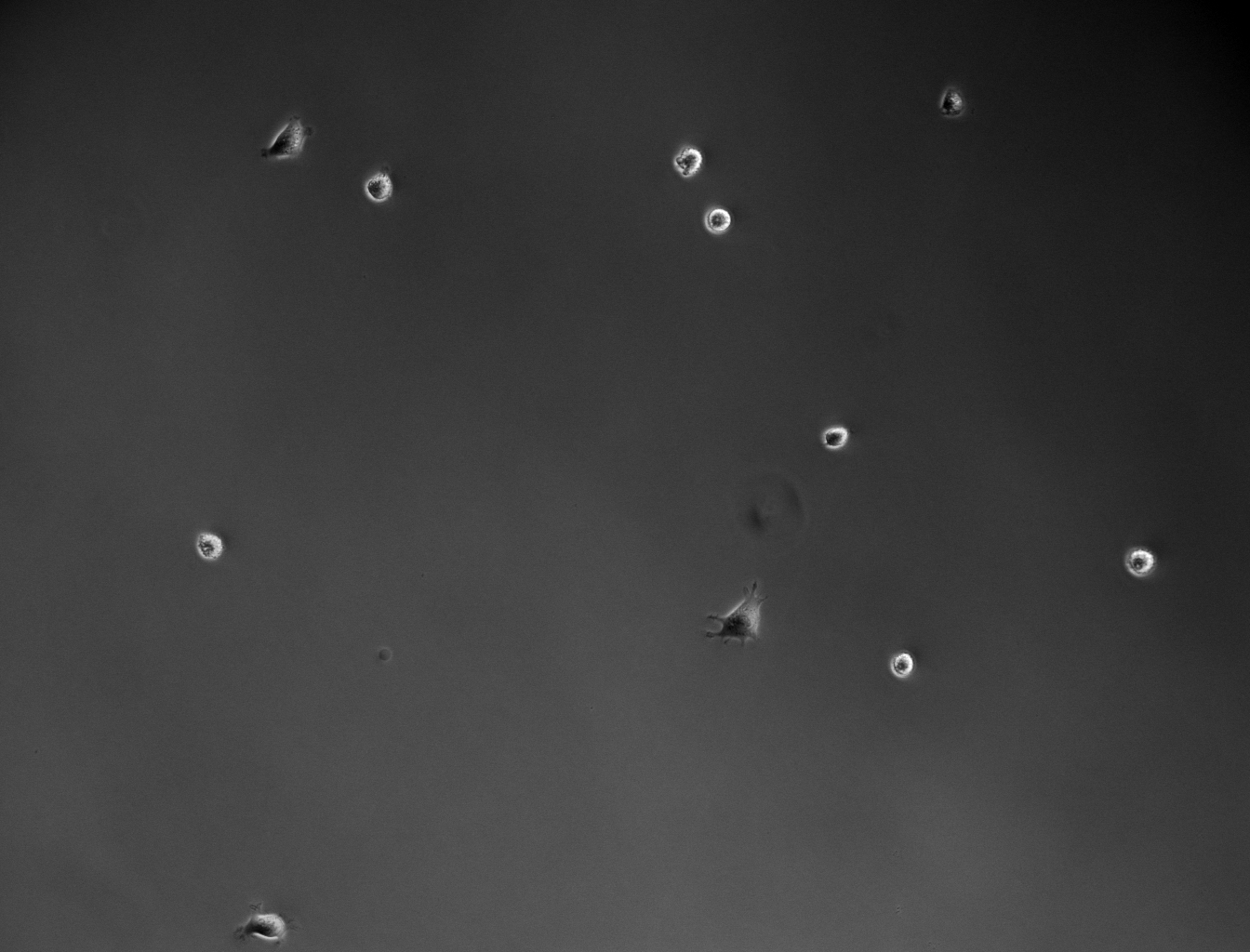

Supplement: Supplementary file 4 — Supplementary Software 1 [file 42003_2022_4117_MOESM4_ESM.zip › SSL_GUI_Demo_Package/Time_Series_Sample_Imagery/MDA_MB_231_Phase_10X_t002.tif]

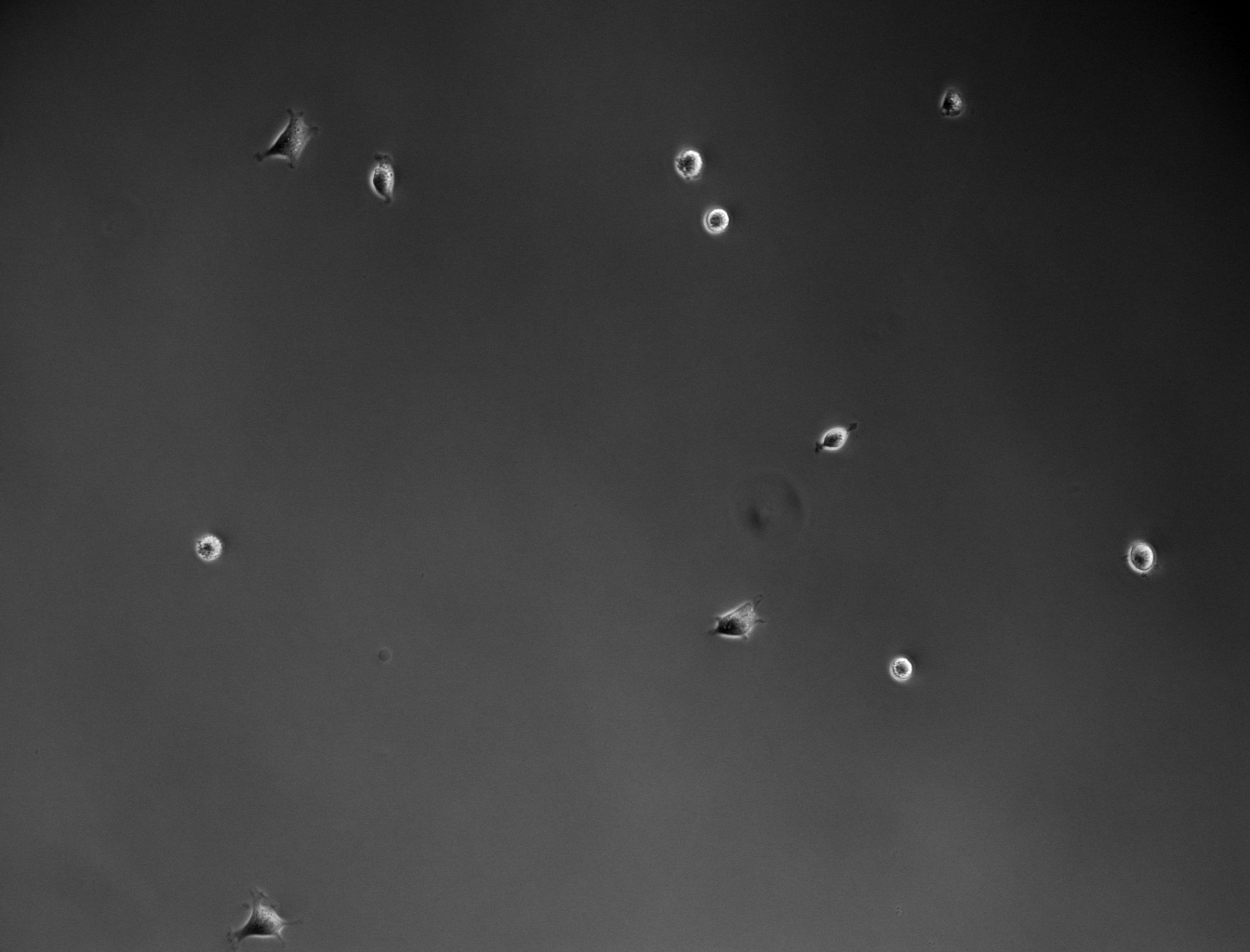

Supplement: Supplementary file 4 — Supplementary Software 1 [file 42003_2022_4117_MOESM4_ESM.zip › SSL_GUI_Demo_Package/Time_Series_Sample_Imagery/MDA_MB_231_Phase_10X_t003.tif]

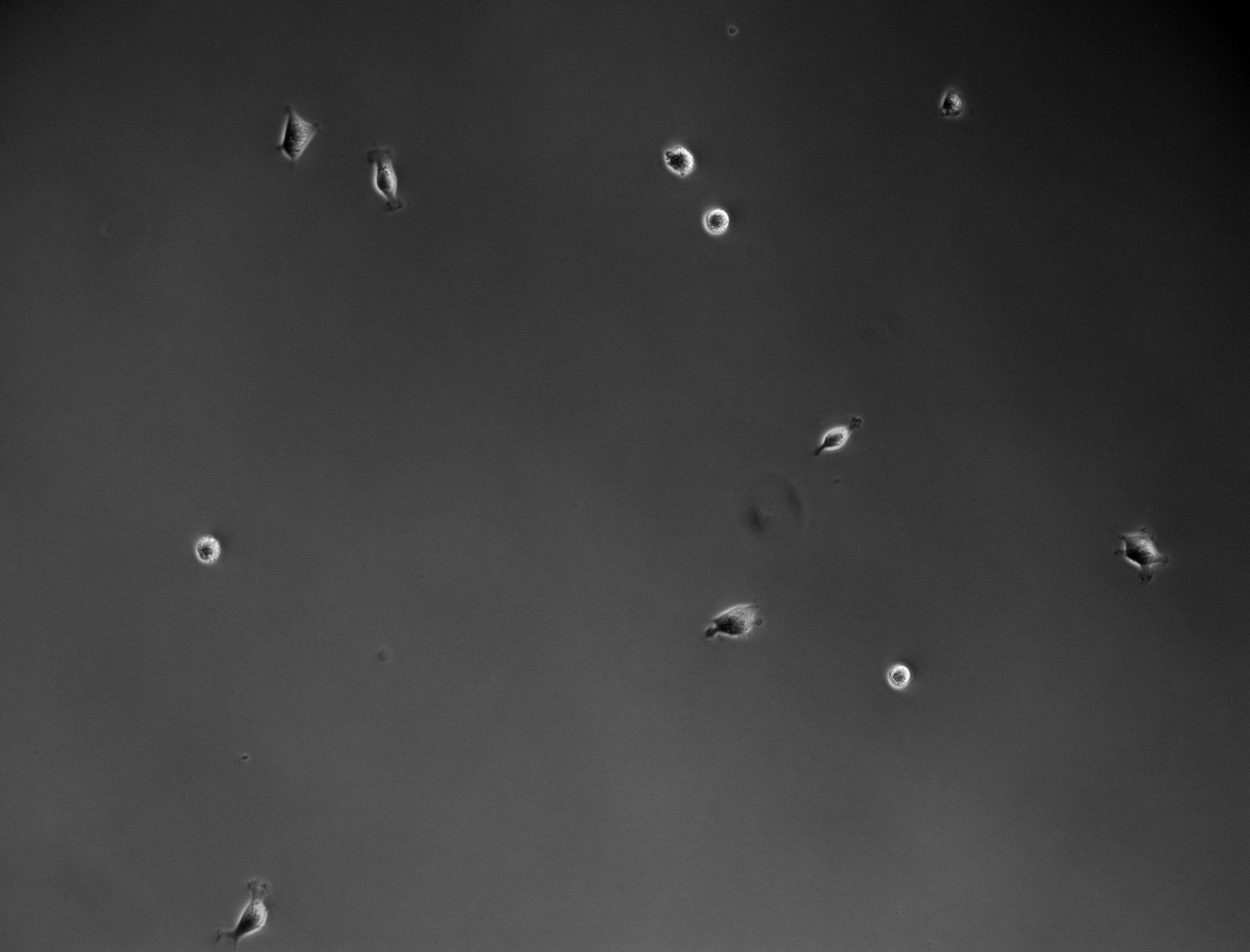

Supplement: Supplementary file 4 — Supplementary Software 1 [file 42003_2022_4117_MOESM4_ESM.zip › SSL_GUI_Demo_Package/Time_Series_Sample_Imagery/MDA_MB_231_Phase_10X_t004.tif]

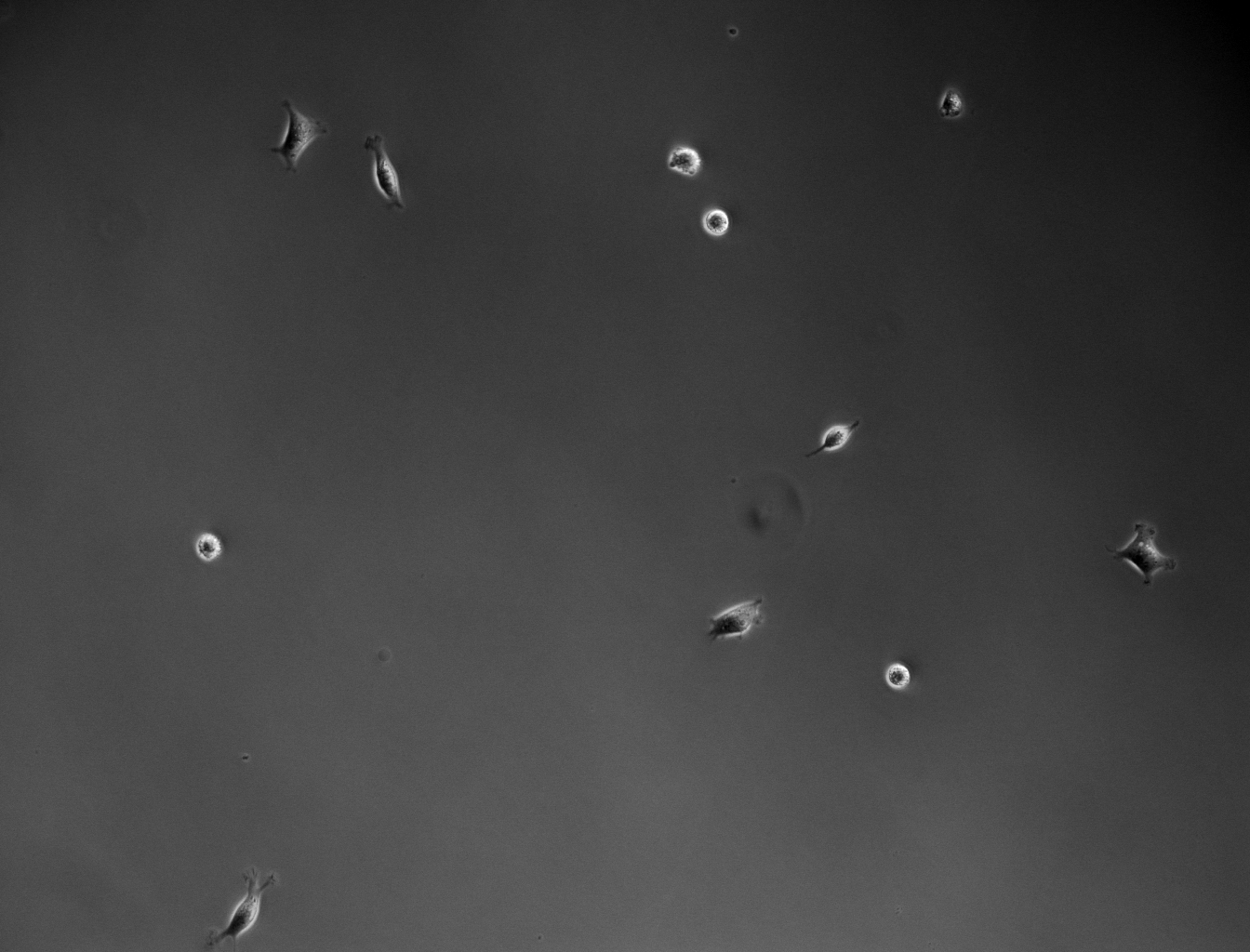

Supplement: Supplementary file 4 — Supplementary Software 1 [file 42003_2022_4117_MOESM4_ESM.zip › SSL_GUI_Demo_Package/Time_Series_Sample_Imagery/MDA_MB_231_Phase_10X_t005.tif]

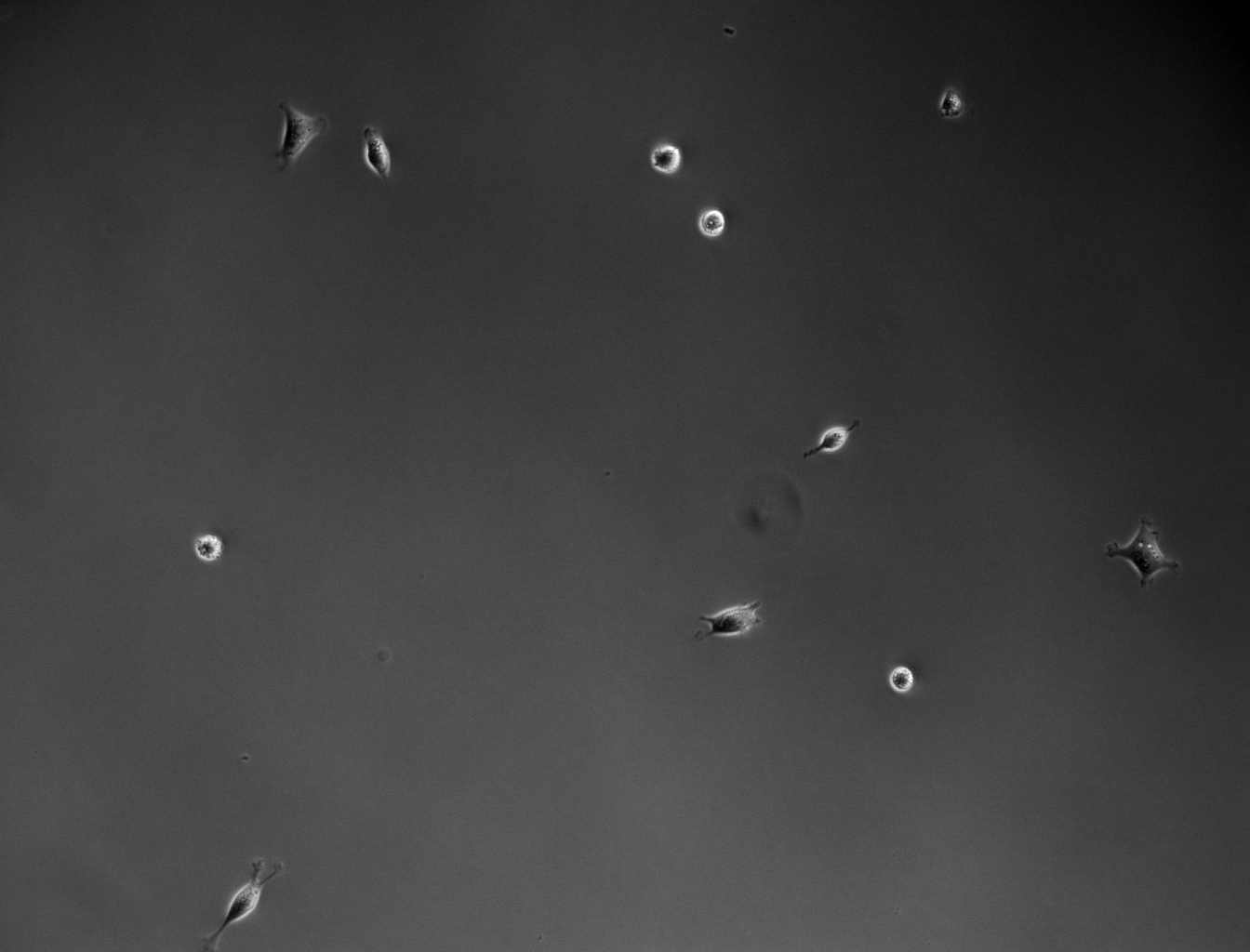

Supplement: Supplementary file 4 — Supplementary Software 1 [file 42003_2022_4117_MOESM4_ESM.zip › SSL_GUI_Demo_Package/Time_Series_Sample_Imagery/MDA_MB_231_Phase_10X_t006.tif]

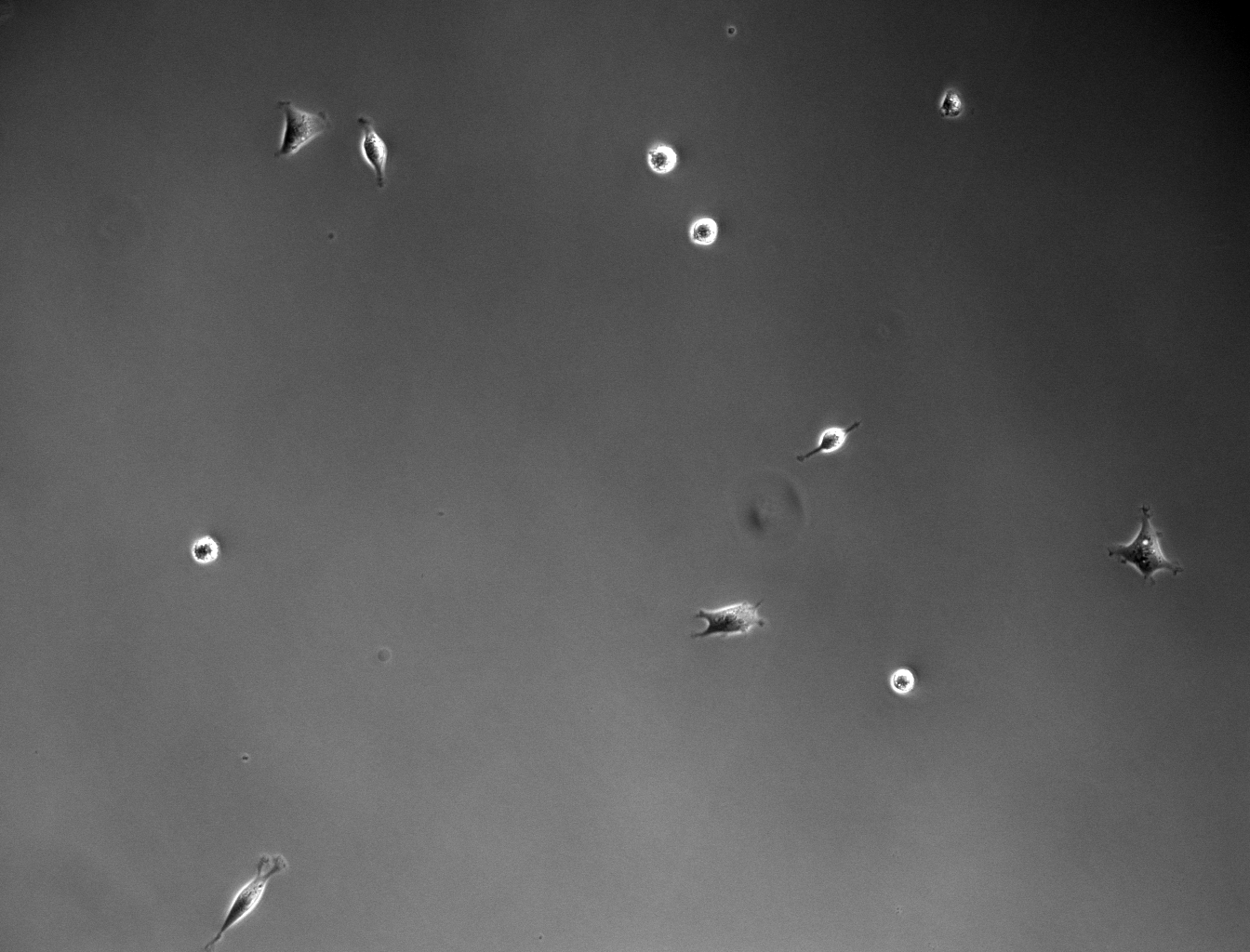

Supplement: Supplementary file 4 — Supplementary Software 1 [file 42003_2022_4117_MOESM4_ESM.zip › SSL_GUI_Demo_Package/Time_Series_Sample_Imagery/MDA_MB_231_Phase_10X_t007.tif]

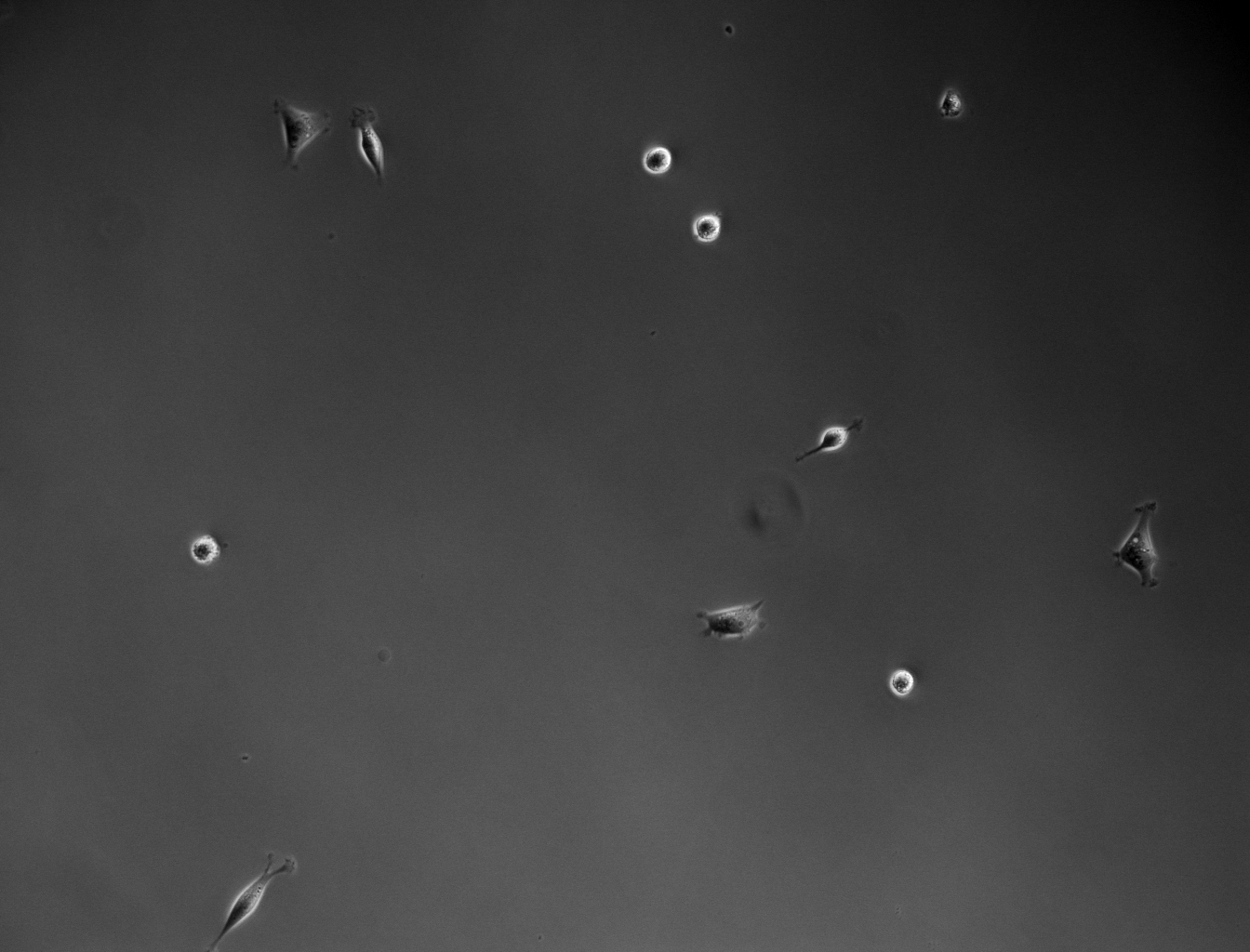

Supplement: Supplementary file 4 — Supplementary Software 1 [file 42003_2022_4117_MOESM4_ESM.zip › SSL_GUI_Demo_Package/Time_Series_Sample_Imagery/MDA_MB_231_Phase_10X_t008.tif]

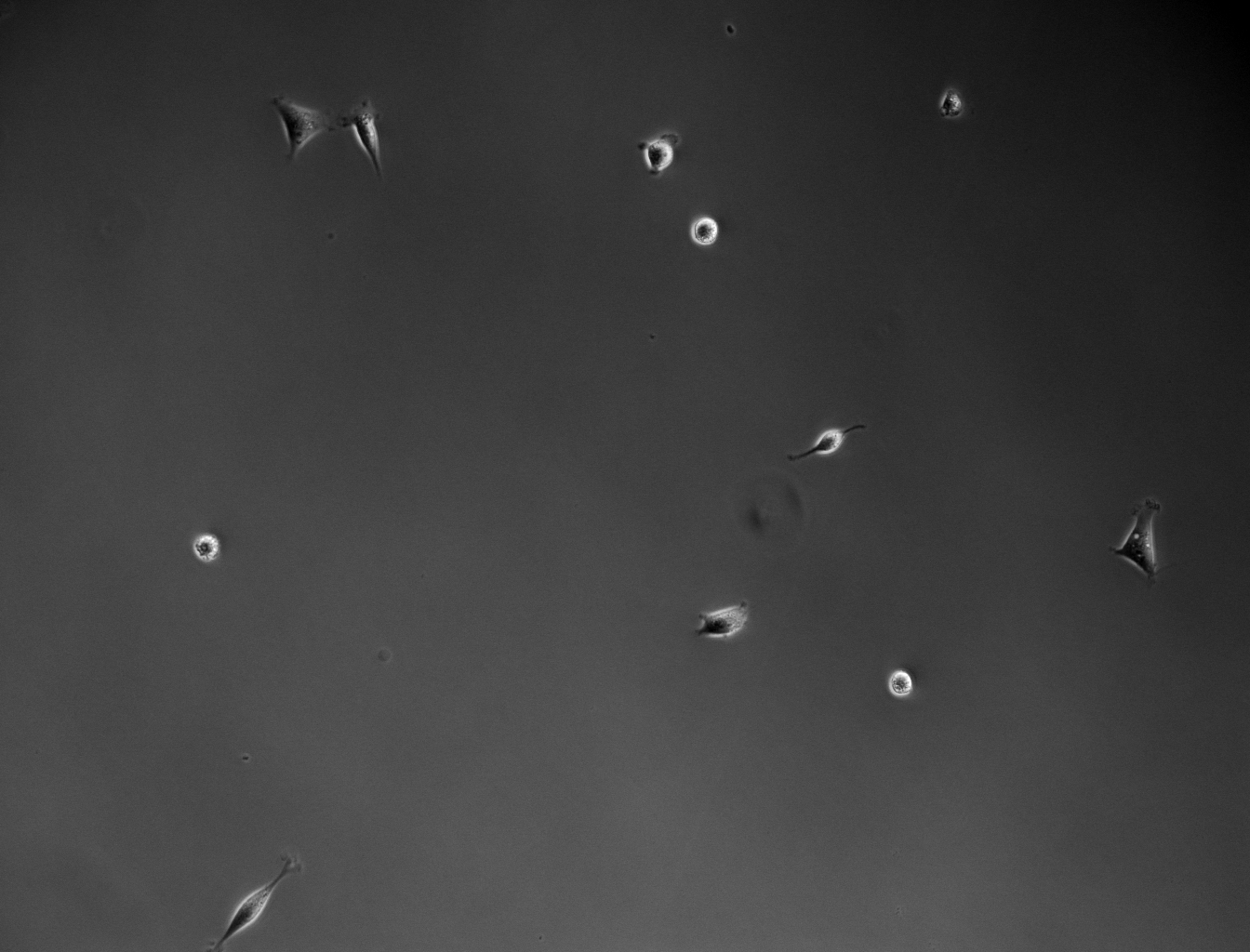

Supplement: Supplementary file 4 — Supplementary Software 1 [file 42003_2022_4117_MOESM4_ESM.zip › SSL_GUI_Demo_Package/Time_Series_Sample_Imagery/MDA_MB_231_Phase_10X_t009.tif]

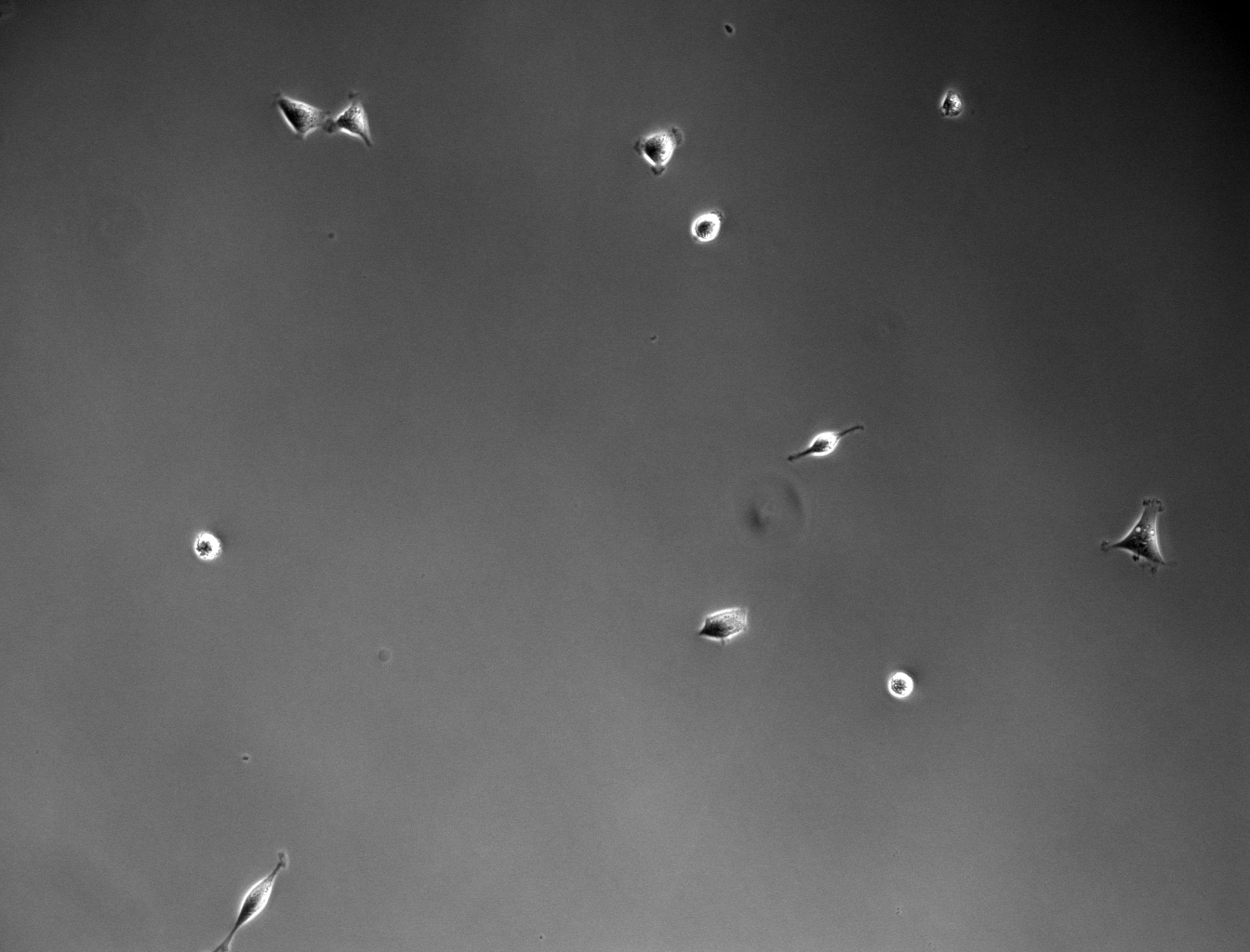

Supplement: Supplementary file 4 — Supplementary Software 1 [file 42003_2022_4117_MOESM4_ESM.zip › SSL_GUI_Demo_Package/Time_Series_Sample_Imagery/MDA_MB_231_Phase_10X_t010.tif]

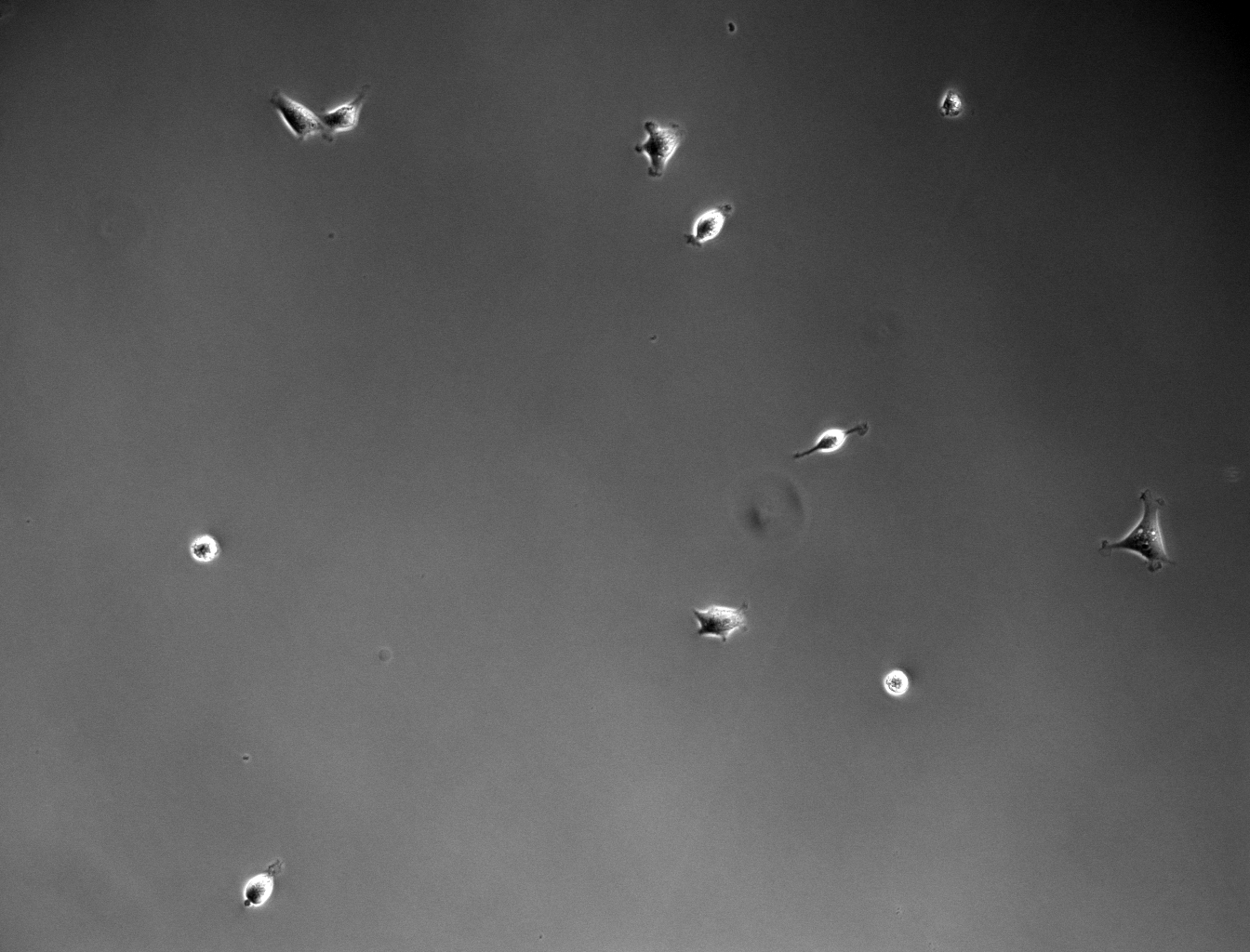

Supplement: Supplementary file 4 — Supplementary Software 1 [file 42003_2022_4117_MOESM4_ESM.zip › SSL_GUI_Demo_Package/Time_Series_Sample_Imagery/MDA_MB_231_Phase_10X_t011.tif]

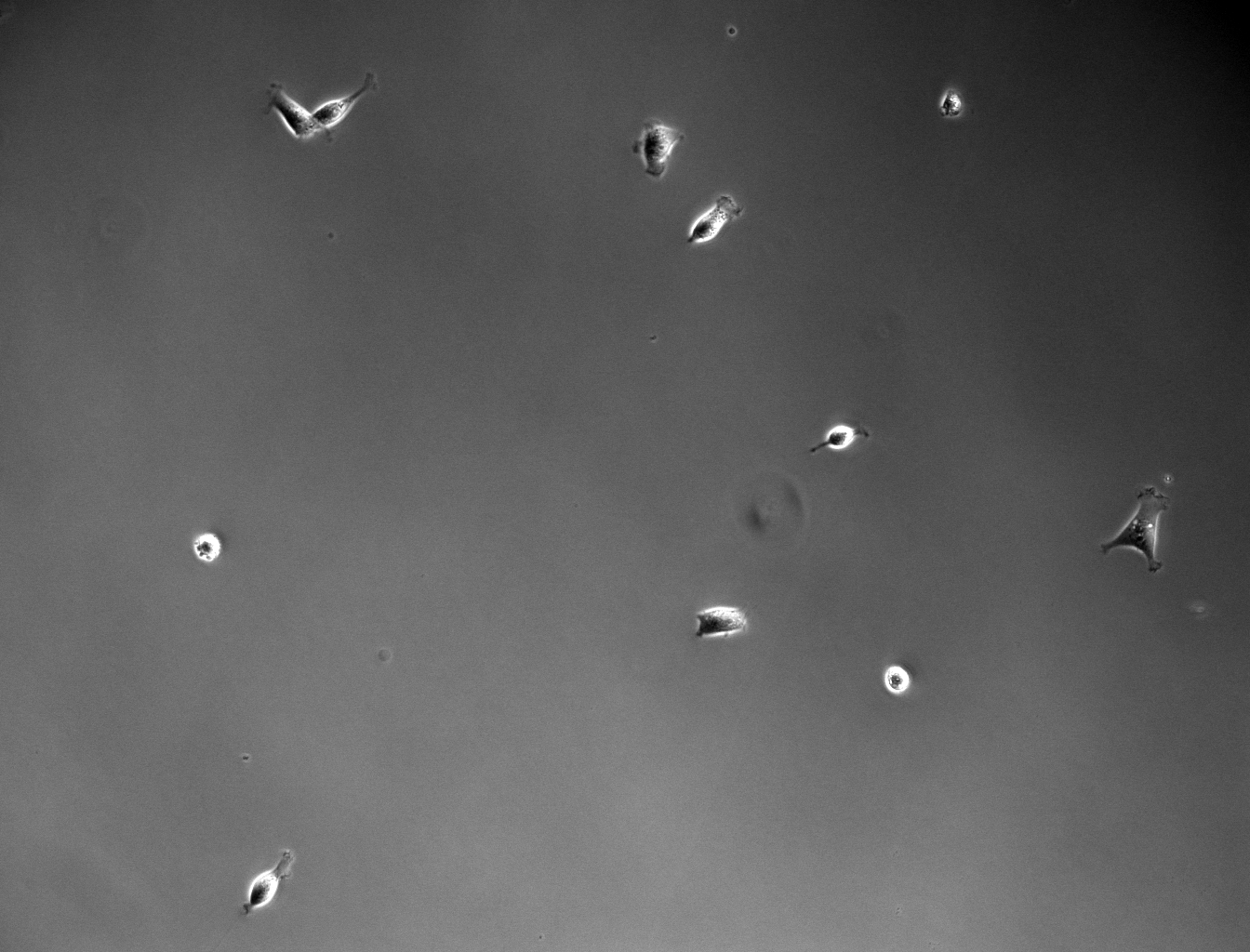

Supplement: Supplementary file 4 — Supplementary Software 1 [file 42003_2022_4117_MOESM4_ESM.zip › SSL_GUI_Demo_Package/Time_Series_Sample_Imagery/MDA_MB_231_Phase_10X_t012.tif]

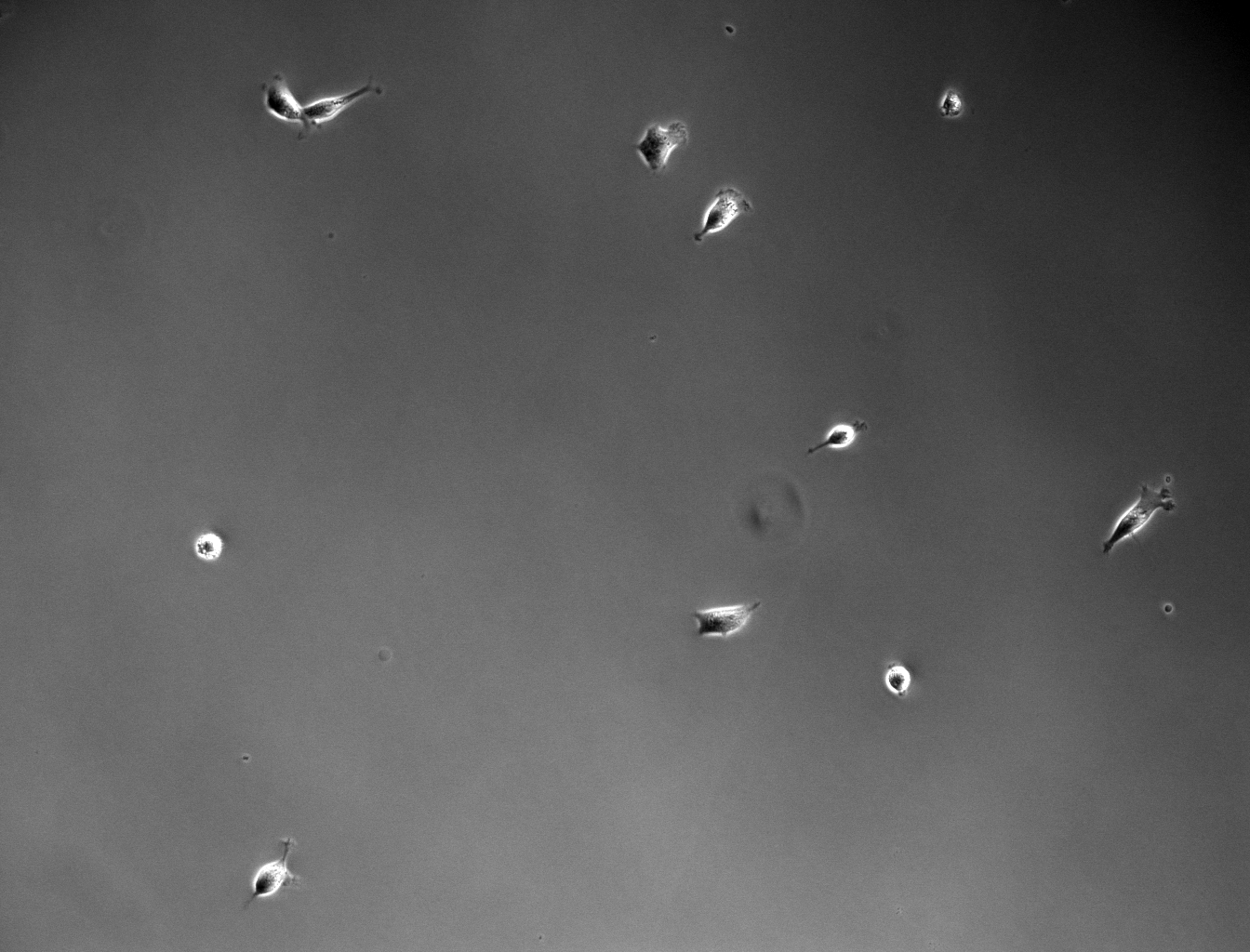

Supplement: Supplementary file 4 — Supplementary Software 1 [file 42003_2022_4117_MOESM4_ESM.zip › SSL_GUI_Demo_Package/Time_Series_Sample_Imagery/MDA_MB_231_Phase_10X_t013.tif]

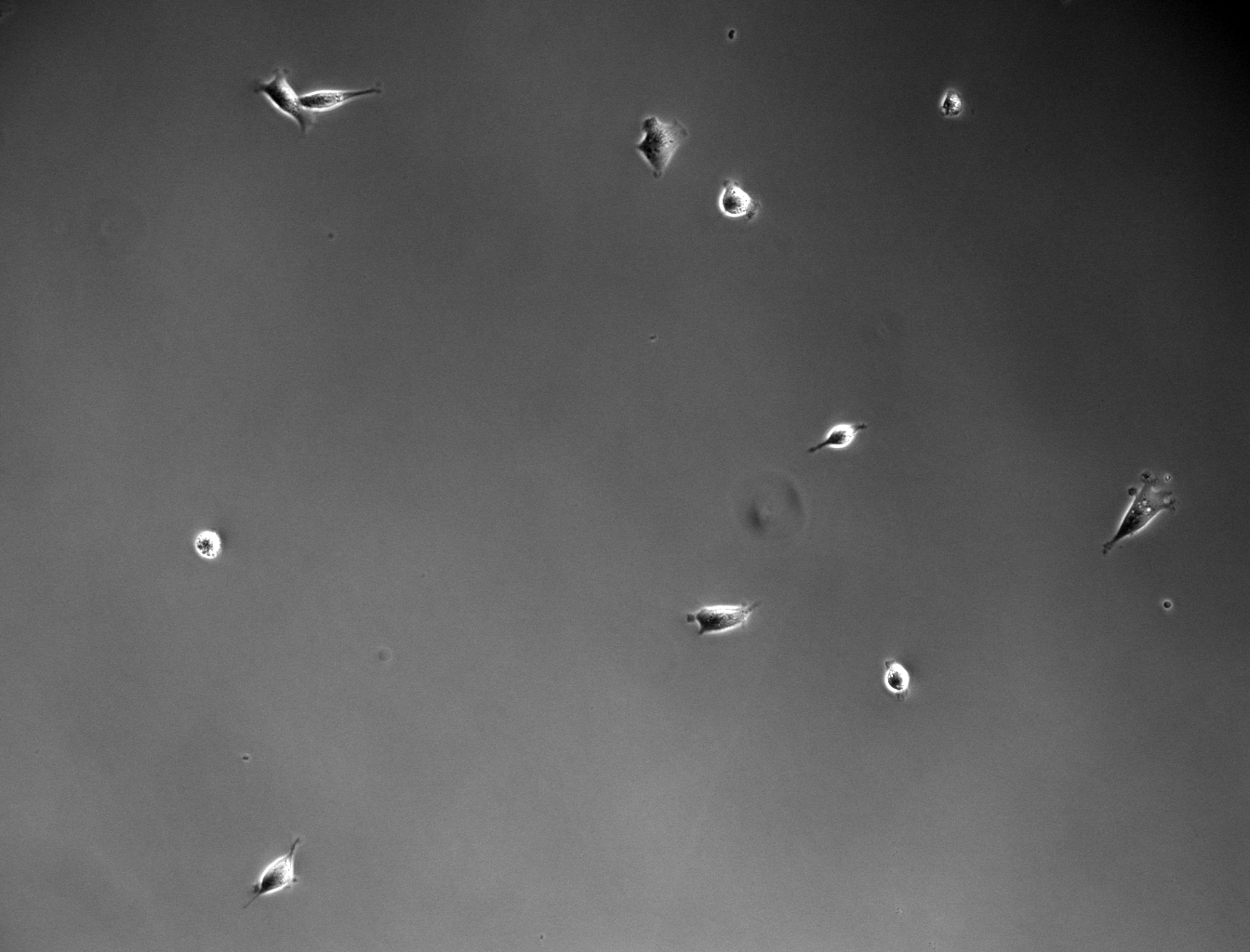

Supplement: Supplementary file 4 — Supplementary Software 1 [file 42003_2022_4117_MOESM4_ESM.zip › SSL_GUI_Demo_Package/Time_Series_Sample_Imagery/MDA_MB_231_Phase_10X_t014.tif]

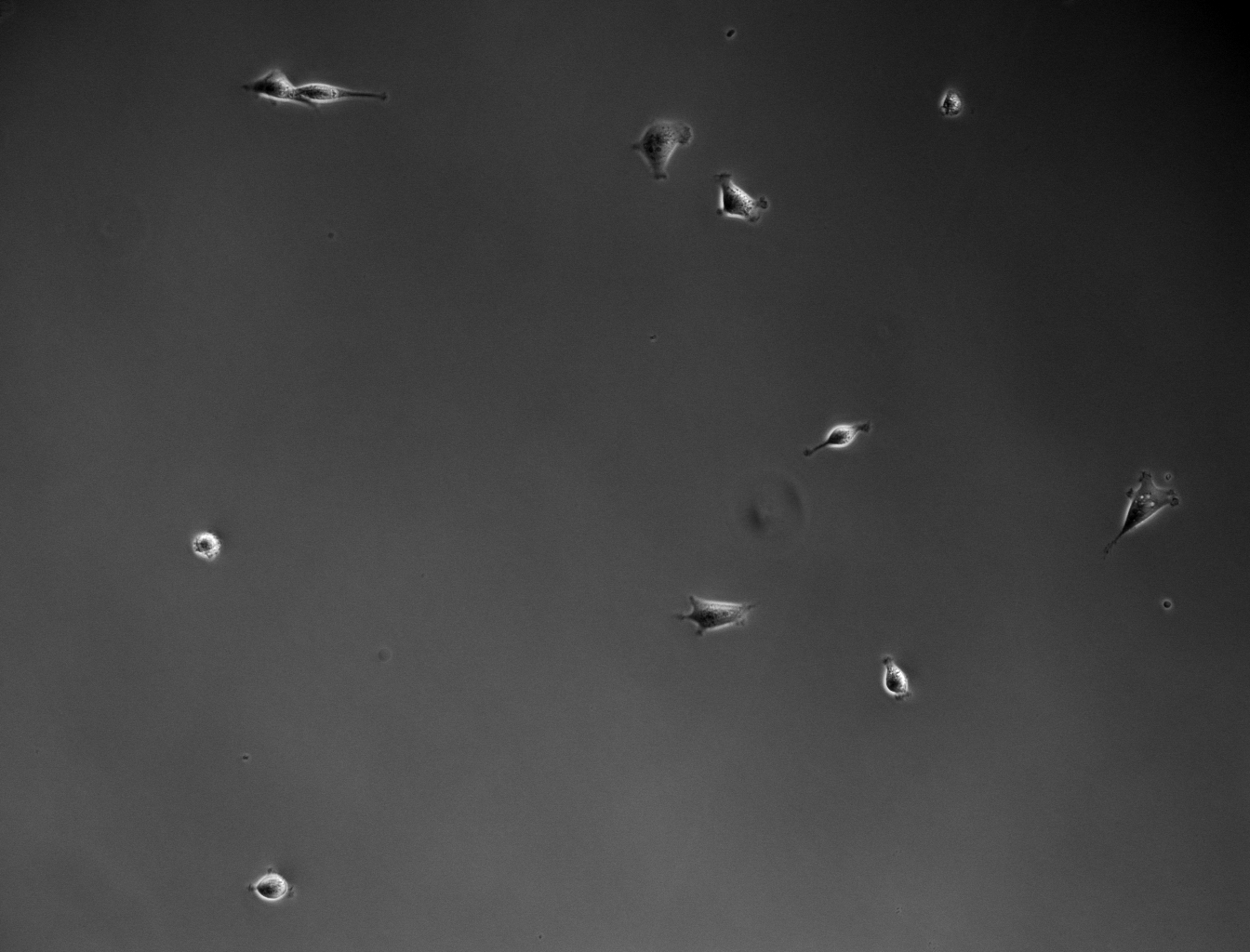

Supplement: Supplementary file 4 — Supplementary Software 1 [file 42003_2022_4117_MOESM4_ESM.zip › SSL_GUI_Demo_Package/Time_Series_Sample_Imagery/MDA_MB_231_Phase_10X_t015.tif]
