# Supplementary material for: Self-supervised machine learning for live cell imagery segmentation
Source: Commun Biol. 2022 Nov 2;5:1162. doi: 10.1038/s42003-022-04117-x (PMC9630527; doi:10.1038/s42003-022-04117-x)
Supplement: Supplementary file 4 — Supplementary Software 1 [file 42003_2022_4117_MOESM4_ESM.zip › SSL_GUI_Demo_Package/GUI_ReadMe.pdf]

GUI Demo Code ReadMe File

Self-supervised machine learning code for segmenting live cell imagery.

The included stand-alone graphical user interfaces (GUIs) are designed to be used with time-resolved live cell microscopy images (tiffs) for the automated segmentation of cells from background. There is a dedicated stand-alone GUI for Windows, Mac and Linux operating systems (OS) :

SSL_Win: Tested on Windows 10 OS
SSL_Mac: Tested on macOS Big Sur Version 11.0.1
SSL_Linux: Tested on Linux Ubuntu 20.04.4 LTS

The GUIs were generated using the Matlab Application Compiler and require Matlab Runtime. A free download of Matlab Runtime can be found here:

https://www.mathworks.com/products/compiler/matlab-runtime.html

Download the 64-bit version zip file associated with your OS of choice using the version indicated by the red boxes below. Extract the zipped files, double click 'setup' and follow the installation instructions.

Release (MATLAB Runtime Version#)	Windows	Linux	Mac
R2022a (9.12)	64-bit	64-bit	Intel 64-bit
R2021b (9.11)	64-bit	64-bit	Intel 64-bit
R2021a (9.10)	64-bit	64-bit	Intel 64-bit

The principle of self-supervised machine learning is that you simply load your images and hit 'Run' - no parameter tuning needed, no training imagery required.

Run from start to finish, the code uses consecutive pairs of images to generate unsupervised training data of 'cells' and 'background' via dynamic feature vectors based on optical flow. These self-labeled pixels are then used to generate static feature vectors (*e.g.* entropy, gradient), which in turn are used to train a classifier model. The training data is updated every image in order to automatically adapt to temporal changes in cell morphologies or background illumination.

This demo code allows the user to reproduce figures from the main manuscript or work with their own images. As an additional example, a short time series of 15 phase contrast images has been included (see 'Time_Series_Sample_Imagery' folder).

To use this code:

1. Unzipped SSL_GUI_Demo_Package
2. Run either SSL_Win, SSL_Mac or SSL_Linux based on your OS
3. Give the GUI several seconds to open (it may take some time for Matlab Runtime to start up in the background)
4. You can select the included imagery from Figure 3 in the manuscript or select at least two images from the "Time_Series_Sample_Imagery" images.

5. You can also add your own data (at least two images) to the \UserData\data folder
6. If the “Select Data from Paper” option gives an error, use the “Select User Data” option and select your tiff files (at least two) from the desired Data folder.
7. Either way, the code will segment the image and create an *.avi movie one directory level above.
8. Note that a Matlab binary mask (*_mask.mat) file of the segmented cells will also be created for future use if desired.
