# Supplementary material for: Self-supervised machine learning for live cell imagery segmentation
Source: Commun Biol. 2022 Nov 2;5:1162. doi: 10.1038/s42003-022-04117-x (PMC9630527; doi:10.1038/s42003-022-04117-x)
Supplement: Supplementary file 5 — Supplementary Software 2 [file 42003_2022_4117_MOESM5_ESM.zip › SSL_Matlab_Demo_Package/ReadMe.pdf]

Matlab Demo Code ReadMe File

Self supervised machine learning code for segmenting live cell imagery.

This Matlab code is designed to be used with time-resolved live cell microscopy images (tiffs) for the automated segmentation of cells from background. This code was tested on Matlab v2020a, v2021a and v2022a using commercially available laptop computers running the Windows 10 operating system. The code requires the following additional toolboxes to be installed:

Computer Vision Toolbox
Image Processing Toolbox
Statistics and Machine Learning Toolbox

Matlab and the associated toolboxes are available for free 30 day trials if not currently licensed by your institution.

The principle of self-supervised machine learning is that you simply load your images and hit 'Run' - no parameter tuning needed, no training imagery required.

Run from start to finish, the code uses consecutive pairs of images to generate unsupervised training data of 'cells' and 'background' via dynamic feature vectors based on optical flow. These self-labeled pixels are then used to generate static feature vectors (*e.g.* entropy, gradient), which in turn are used to train a classifier model. The training data is updated every image in order to automatically adapt to temporal changes in cell morphologies or background illumination.

This demo code allows the user to reproduce figures from the main manuscript or work with their own images. As an additional example, a short time series of 15 phase contrast images has been included (see 'Time_Series_Sample_Imagery' folder).

To use this code:

1. Place the unzipped SSL_Demo_Package folder in your Matlab working directory
2. Open SSL_Demo_NC.m
3. Run the code
4. When prompted in the command window, answer whether or not you want to reproduce a figure from the paper.
5. If yes, you will be prompted for the Fig 3 letter (a, b, c…)
6. If no, you will be prompted to first place your tiff imagery (or the included time series sample imagery) into the SSL_Demo_Package\UserData\data folder
7. Either way, the code will segment the imagery, place a binary mask of the segmented cells in the associated 'mask' folder and create an *.avi movie one directory level above.
