# Supplementary material for: Self-supervised machine learning for live cell imagery segmentation
Source: Commun Biol. 2022 Nov 2;5:1162. doi: 10.1038/s42003-022-04117-x (PMC9630527; doi:10.1038/s42003-022-04117-x)
Supplement: Supplementary file 6 — Reporting Summary [file 42003_2022_4117_MOESM6_ESM.pdf]

nature research

Corresponding author(s): Marc P. Raphael

Last updated by author(s): 9/15/2022

Reporting Summary

Nature Research wishes to improve the reproducibility of the work that we publish. This form provides structure for consistency and transparency in reporting. For further information on Nature Research policies, see our Editorial Policies and the Editorial Policy Checklist.

Statistics

For all statistical analyses, confirm that the following items are present in the figure legend, table legend, main text, or Methods section.

n/a	Confirmed	
☐	☒	The exact sample size (*n*) for each experimental group/condition, given as a discrete number and unit of measurement
☐	☒	A statement on whether measurements were taken from distinct samples or whether the same sample was measured repeatedly
☒	☐	The statistical test(s) used AND whether they are one- or two-sided<br>*Only common tests should be described solely by name; describe more complex techniques in the Methods section.*
☒	☐	A description of all covariates tested
☒	☐	A description of any assumptions or corrections, such as tests of normality and adjustment for multiple comparisons
☒	☐	A full description of the statistical parameters including central tendency (e.g. means) or other basic estimates (e.g. regression coefficient) AND variation (e.g. standard deviation) or associated estimates of uncertainty (e.g. confidence intervals)
☒	☐	For null hypothesis testing, the test statistic (e.g. F, t, r) with confidence intervals, effect sizes, degrees of freedom and P value noted<br>*Give P values as exact values whenever suitable.*
☐	☒	For Bayesian analysis, information on the choice of priors and Markov chain Monte Carlo settings
☒	☐	For hierarchical and complex designs, identification of the appropriate level for tests and full reporting of outcomes
☒	☐	Estimates of effect sizes (e.g. Cohen's d, Pearson's r), indicating how they were calculated

Our web collection on statistics for biologists contains articles on many of the points above.

Software and code

Policy information about availability of computer code

Data collection	All data was collected using Zen Blue (Carl Zeiss Microscopy, LLC) image acquisition software version
Data analysis	All code was written in Matlab (The Mathworks, Inc) and tested on versions R2020a and R2021a

For manuscripts utilizing custom algorithms or software that are central to the research but not yet described in published literature, software must be made available to editors and reviewers. We strongly encourage code deposition in a community repository (e.g. GitHub). See the Nature Research guidelines for submitting code & software for further information.

Data

Policy information about availability of data

All manuscripts must include a data availability statement. This statement should provide the following information, where applicable:

- Accession codes, unique identifiers, or web links for publicly available datasets
- A list of figures that have associated raw data
- A description of any restrictions on data availability

All code and imagery to be made available as supplemental information

Field-specific reporting

Please select the one below that is the best fit for your research. If you are not sure, read the appropriate sections before making your selection.

☒ Life sciences ☐ Behavioural & social sciences ☐ Ecological, evolutionary & environmental sciences

For a reference copy of the document with all sections, see nature.com/documents/nr-reporting-summary-flat.pdf

Life sciences study design

All studies must disclose on these points even when the disclosure is negative.

Sample size	N=2 for shown images (The algorithm self-supervises using pairs of consecutive images within a given image time stack)
Data exclusions	Not applicable for this computational study
Replication	The algorithm was tested on 5 different cell types (from amoeboid to mammalian) using 5 different microscopy modes (fluorescence, DIC, phase, IRM, transmitted light) to ensure robustness.
Randomization	Not applicable for this computational study
Blinding	All analysis is inherently blinded in the self supervised ML approach because the user cannot influence the results by training or tuning.

Reporting for specific materials, systems and methods

We require information from authors about some types of materials, experimental systems and methods used in many studies. Here, indicate whether each material, system or method listed is relevant to your study. If you are not sure if a list item applies to your research, read the appropriate section before selecting a response.

Materials & experimental systems

n/a	Involved in the study	
☒	☐	Antibodies
☐	☒	Eukaryotic cell lines
☒	☐	Palaeontology and archaeology
☒	☐	Animals and other organisms
☒	☐	Human research participants
☒	☐	Clinical data
☒	☐	Dual use research of concern

Methods

n/a	Involved in the study	
☒	☐	ChIP-seq
☒	☐	Flow cytometry
☒	☐	MRI-based neuroimaging

Eukaryotic cell lines

Policy information about cell lines

Cell line source(s)	All cell lines were purchased from ATCC except for Dictyostelium discoideum which was a gift from the Devreotes laboratory of Johns Hopkins University
Authentication	All ATCC purchased cell lines came with certificates of authenticity. Details on Dictyostelium discodium authentication from the Devreotes laboratory at Johns Hopkins University can be found in the following publication: Miao, Y., Bhattacharya, S., Edwards, M. et al. Altering the threshold of an excitable signal transduction network changes cell migratory modes. Nat Cell Biol 19, 329–340 (2017). https://doi.org/10.1038/ncb3495
Mycoplasma contamination	Cell lines not tested regularly for mycloplasma contamination because this is a computational study.
Commonly misidentified lines (See ICLAC register)	None
